# Diversity in Compartmental Dynamics of Gene Regulatory Networks: The Immune Response in Primary Influenza A Infection in Mice

**DOI:** 10.1371/journal.pone.0138110

**Published:** 2015-09-28

**Authors:** Xing Qiu, Shuang Wu, Shannon P. Hilchey, Juilee Thakar, Zhi-Ping Liu, Stephen L. Welle, Alicia D. Henn, Hulin Wu, Martin S. Zand

**Affiliations:** 1 Department of Biostatistics and Computational Biology, University of Rochester Medical Center, Rochester, NY, 14642, United States of America; 2 Department of Medicine, University of Rochester Medical Center, Rochester, NY, 14642, United States of America; 3 Department of Microbiology and Immunology, University of Rochester, Rochester, NY, 14642 United States of America; 4 Department of Biomedical Engineering, Shandong University, Jinan, Shandong, China; 5 Functional Genomics Center, University of Rochester, Rochester, NY, 14642, United States of America; 6 Department of Biostatistics, School of Public Health, University of Texas Health Science Center at Houston, Houston, TX, 77030, United States of America; University of Alabama at Birmingham, UNITED STATES

## Abstract

Current approaches to study transcriptional profiles post influenza infection typically rely on tissue sampling from one or two sites at a few time points, such as spleen and lung in murine models. In this study, we infected female C57/BL6 mice intranasally with mouse-adapted H3N2/Hong Kong/X31 avian influenza A virus, and then analyzed the gene expression profiles in four different compartments (blood, lung, mediastinal lymph nodes, and spleen) over 11 consecutive days post infection. These data were analyzed by an advanced statistical procedure based on ordinary differential equation (ODE) modeling. Vastly different lists of significant genes were identified by the same statistical procedure in each compartment. Only 11 of them are significant in all four compartments. We classified significant genes in each compartment into co-expressed modules based on temporal expression patterns. We then performed functional enrichment analysis on these co-expression modules and identified significant pathway and functional motifs. Finally, we used an ODE based model to reconstruct gene regulatory network (GRN) for each compartment and studied their network properties.

## Introduction

Seasonal influenza infection affects 1 billion people annually, causing up to 500,000 deaths each year [1]. The host immune response to infection involves multiple tissue compartments, including the respiratory tract, peripheral blood, regional lymph nodes, and the spleen [2–4]. Migration of immune cells between compartments is critical for establishing effective T and B cell mediated immune responses, and creating adaptive immune memory as a protection against further infection [5–8]. Within each tissue compartment, affected and responding cells (e.g. CD4 and CD8 T cells, respiratory endothelium, B cells) exhibit different phenotypic and functional activities. These compartment-specific activities also vary over the time-frame of the immune response [3, 8–13]. Thus, understanding the dynamic patterns of gene expression within each compartment, how they are linked and how they are temporally and geographically domain specific, is critical to a systems biology understanding of the host immune response to influenza.

Current approaches to study transcriptional profiles post influenza infection typically rely on tissue sampling from one or two sites, generally spleen and lung in murine models, and these samples are often collected at only a few time points. This approach, however, offers only a limited snapshot of transcriptional changes throughout the course of infection. In contrast, comprehensive understanding of whole compartment transcriptome variations after infection has provided valuable insights into location-specific changes after HIV, transmissible spongiform encephalitis (TSE) [14], *Francisella tularensis* [15], and avian pathogenic *Escherichia coli* (APEC) [16] infections. Global transcriptome analysis has been reported for whole lung in a murine infection model, but without comparison to regional lymph node, peripheral blood, and spleen [17]. Such multi-compartment information is critical when bridging the gap between murine studies of the influenza immune response, where we can sample multiple tissue compartments, and human studies, where sampling is limited to peripheral blood and perhaps lung.

To address this issue, we studied the dynamic immune responses to influenza infection at the transcriptional level by simultaneous daily sampling of lymphocytes in four different compartments (blood, lung, mediastinal lymph nodes, and spleen) over 11 consecutive days post infection. Data were analyzed with a procedure based on high-dimensional ordinary differential equation (ODE) models [18] to reconstruct gene regulatory networks (GRNs). We found that the four compartments exhibit wide variation in gene expression patterns, with the number and identity of differentially expressed genes being very different between compartments. Clustering analysis of differentially expressed genes by their temporal expression patterns also showed marked differences in the time to increased or decreased expression in each compartment, allowing us to observe and analyze the temporal sequence of a global “transcriptome cascade” between compartments. In addition, gene set enrichment analyses show that the functional annotations of the clusters have different enriched terms and the network (edges) between these nodes are very different. The prevalence of delayed genes in the lung highlights the importance of understanding cellular trafficking kinetics in the immune response to influenza infection. Our findings suggest that: a) Compartment specific transcriptomes are regulated by very different networks in different compartments; and b) Using temporal gene expression data by frequent sampling can reveal the dynamic features of gene regulatory networks, which are hard to detect from cross-sectional data.

## Results

### Experimental System Summary

Female C57/BL6 mice were infected intranasally with a mouse-adapted H3N2/Hong Kong/X31 avian influenza A virus [19]. Infected mice were sacrificed in groups of five daily, from t = 0 DPI (days post-infection) to t = 10 DPI. Four compartments (lung, mediastinal lymph node, blood, and spleen) were collected from each mouse and pooled into three groups to measure genome-wide gene expression using Affymetrix® Mouse Gene 1.0 ST RNA microarrays. We reconstructed the gene regulatory networks (GRNs) using a recently developed statistical procedure [18]. This procedure can be roughly divided into three steps: 1) identifying temporally differentially expressed genes (TDEGs); 2) clustering the identified TDEGs into gene modules based on features of their temporal expression patterns; 3) reconstructing the gene regulatory network by a computational framework based on ordinary differential equations. In each step, the results are processed by gene set enrichment analyses to reveal more information relevant to the underlying immune-response process.

### Temporally Differentially Expressed Genes

We selected temporally differentially expressed probe sets from each compartment using functional principal component analysis [20]. The number of significant probe sets varies significantly between compartments: 1,906 in lung; 3,548 in lymph node; 224 in blood; and 508 in spleen. While the large differences between the numbers of significant probe sets identified in each compartment are in part due to the different signal-to-noise ratios of gene expression (please see Fig A in S1 Text for more details), this analysis also demonstrates that influenza infection affects each compartment very differently at the level of differential expression. One goal of this study is to analyze the differences in genomic responses to influenza infection at higher levels, such as clusters, reconstructed GRNs, and enriched functions. To this end, we analyzed the top 1000 most significant probe sets from the blood and spleen compartment for the subsequent analysis. This ensured that the observed high level differences in transcriptome membership and patterns were not merely a consequence of the variable numbers of significant probe sets identified between compartments. The corresponding relaxed significance levels were 0.0049 for unadjusted *p*-values or 0.1445 for Benjamini-Hochberg adjusted *p*-values for the spleen compartment; 0.0032 for unadjusted *p*-values or 0.0964 for Benjamini-Hochberg adjusted *p*-values for the blood compartment. The selected probe sets were then mapped to National Center for Biotechnology Information (NCBI) gene names, and collapsed to single gene names by selecting one unique probe that had the largest inter-quantile range (IQR).

**Fig 1:**
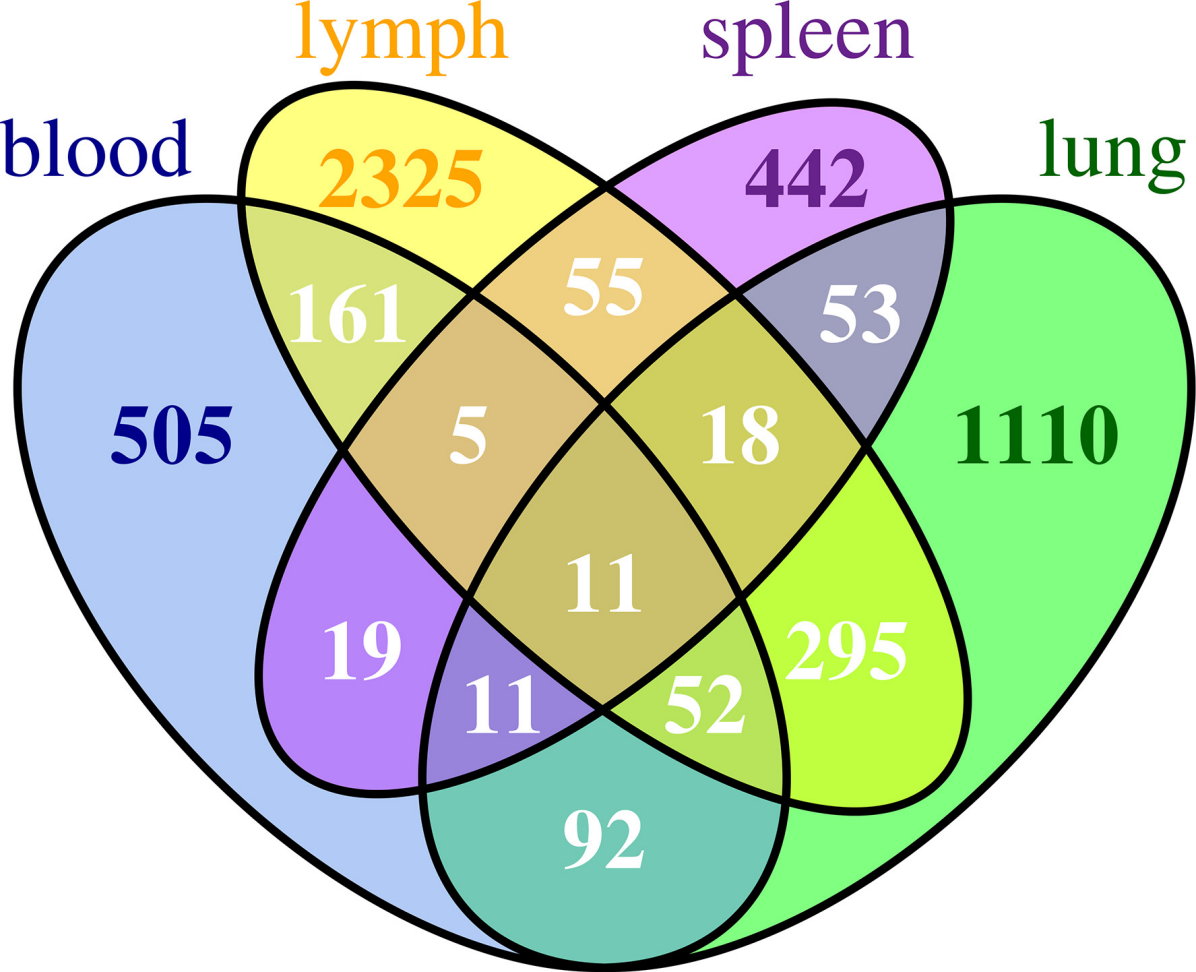
A Venn diagram showing the number of common TDEGs shared by all compartments.

After mapping, the numbers of temporally differentially expressed genes (TDEGs) in each compartment were: 1,642 in lung; 2,922 in lymph node; 856 in blood; and 614 in spleen. Among them, only 8 genes (Ddx60, Ehd4, Gvin1, Ly6a, Ly6c2, Ms4a4c, Phf11 and Xaf1) and 3 pseudo-genes (Gm1966, Gm6545, Gm7609) were common to all compartments. On average, only about 10% of TDEGs were shared by two compartments. Fig 1 is a Venn diagram illustrating the number of common TDEGs shared by multiple compartments. Detailed information about the known biological functions associated with these genes is summarized in S1 Table.

Of particular interest is the up-regulation of Ddx60, a DEXD/H box RNA helicase activated in the cellular response to foreign RNA or DNA [21]. Ddx60 interferes with viral replication, and is also a positive regulator of the MDA-5 and RIG-I signaling pathways, which in turn up-regulate Type I interferon and directly interfere with influenza virus replication [22, 23]. Broad compartmental expression of Ddx60 found here is the first suggestion that it may be systemically induced as an innate immune defense mechanism after influenza infection. In addition to Ddx60, Phf11, a positive regulator of Th1-type cytokine gene expression, including the cytokines IL-2 and IFN-γ, is also upregulated in all four compartments [24]. The genes Ly6a and Ly6c2, markers of T and NK cell activation, also had broadly altered expression across all four compartments after influenza infection [25, 26]. Xaf1, XIAP associated factor 1, is involved in apoptosis, in particular mediating TNF-α-induced apoptosis and has been shown to be upregulated by Type 1 interferons [27]. Gvin1 encodes interferon-induced very large GTPase 1 and belongs to a class of interferon inducible genes involved in cell autonomous immunity [28]. Ehd4 is a member of the C-terminal Eps15 homology domain (EHD) protein family that has been shown to be involved in early endosomal transport. As the knock down of Ehd4 results in the accumulation of MHC-I within the early endosome, preventing recycling back to the cell surface [29], Ehd4 may play a role in the accumulation of extracellular viral particles and cycling through the lysosome for presentation of influenza antigens in the context of MHC-I. This would allow recognition of virus-infected cells by CD8 T cells, and aid in the adaptive immune response. Ms4a4c belongs to the membrane-spanning 4-domains, subfamily A gene cluster (MS4A) and has been shown to be a glucocorticoid induced TNF receptor (GITR) associated membrane adapter that augments GITR signaling and IL-2 production by T cells, resulting in enhanced T cell sensitivity to extrinsic antigen [30].

While we expected a fraction of significant genes to be common between compartments, the extremely low number suggests that common transcriptome response in different compartments after influenza infection is rare. Furthermore, the temporal patterns for these common TDEGs are quite different in the four compartments (Figs B-E in S1 Text). For example, the expressions of these common TDEGs change more gradually in lung than in other compartments. In the lymph node compartment, the expressions of all these genes increase sharply on Day 1, which is a feature not shared by other compartments. Of note, the overall temporal patterns of TDEGs, such as the median activation time and peak time, are different in different compartments. This observation has practical implications for experimental design and will be discussed in the Discussion Section.

### Gene Modules Identified by Temporal Expression Patterns

**Fig 2:**
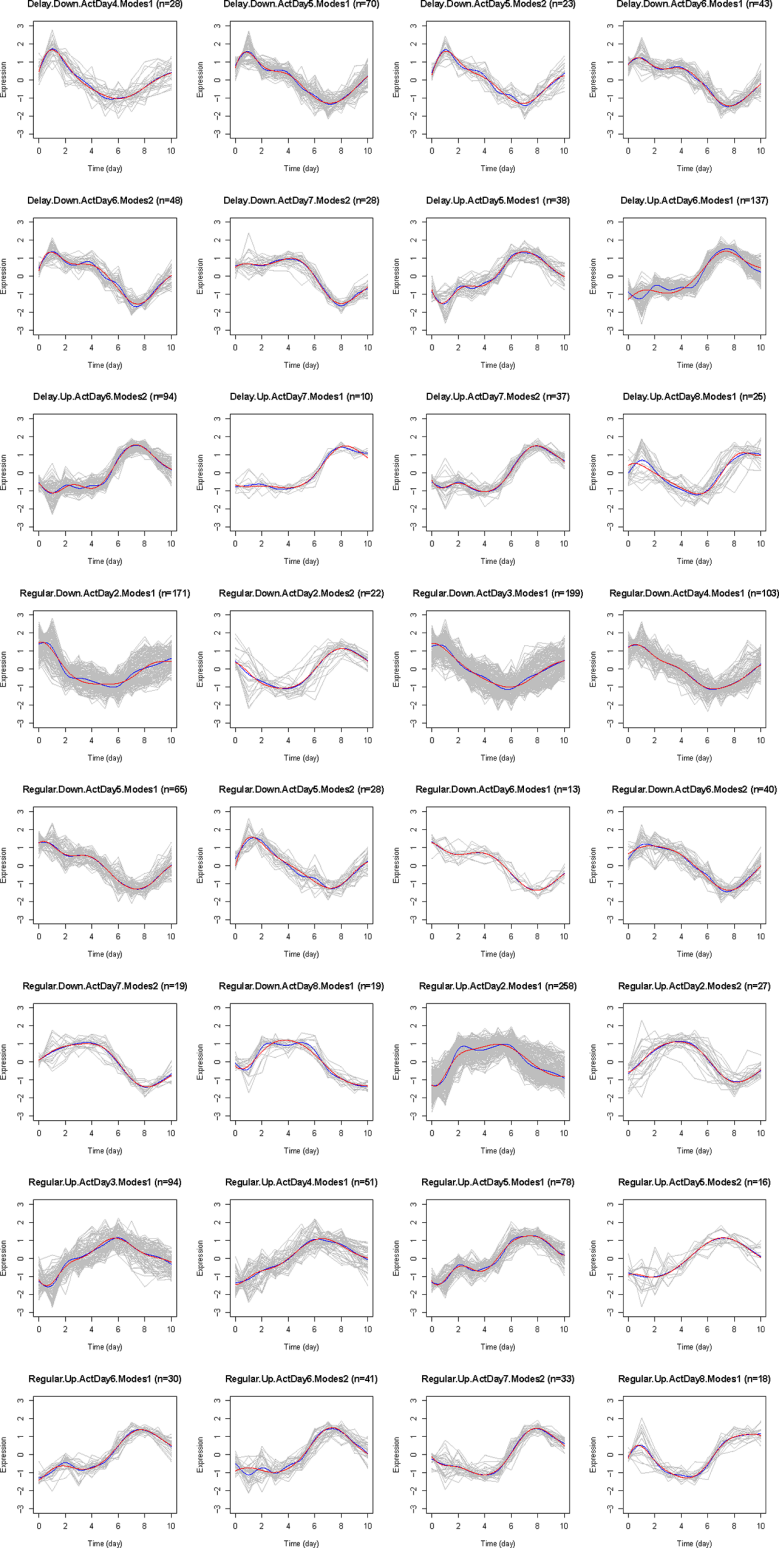
The line plots of each cluster in the lung compartment. TDEGs are classified into co-expression modules. Grey curves represent the temporal trajectory of expression levels for each gene, standardized to zero mean and unit standard deviation. Blue curves are the smoothed mean expression levels for each cluster. Red curves are the predicted mean expression levels from the ODE model. Each co-expression module is classified by four criteria: Delay or Regular; Up or Down; the activation day of its mean gene expression; and number of modes of its mean expression. This information is shown in the subtitles, together with the numbers of genes contained in these modules.

We next classified TDEGs identified in each compartment into temporally co-expressed gene modules based on four temporal expression pattern characteristics: early versus delayed transcription activation, the day of activation, up or down regulation, and the number of modes (local minima/maxima). We used feature based clustering to map the temporal features of the data to biologically relevant functions. Details of the criteria and method can be found in the Materials and Methods Section. Using feature based clustering, we identified multiple unique clusters in each compartment: 12 (blood), 32 (lung), 24 (lymph node), and 16 (spleen), shown in Fig 2 and Figs F-H in S1 Text. Judged by the number of clusters of each compartment, TDEGs in lung have the most diverse temporal patterns and TDEGs in blood are least diverse, the significance of which were next explored with gene set enrichment analysis (S2–S5 Tables).

To perform gene set enrichment analysis, we first clustered genes into two classes: whether there is a time delay, or not, for the transcript levels to increase or decrease compared to the baseline. Genes with a transcription-change time delay are referred to as “delayed genes”, and those without as “early genes". Based on this criterion, 511 and 49 genes are identified as delayed genes for the lung and lymph node, respectively. In contrast, no delayed genes were identified in blood and spleen samples. Fig I in S1 Text shows some typical temporal expression patters for delayed genes in the lung compartment. The above finding suggested that we could identify compartment-specific temporal changes in the character of the immune response and functions of early and delayed expression genes. Thus, we next performed transcriptome functional enrichment analyses, within and across compartments, to identify specific pathway and functional motifs present in each gene module.

### Transcriptome Changes between the Early and Delayed Clusters Suggest a Switch between the Innate and Adaptive Immune Responses in Lung

In our previous work [8], computational modeling of murine influenza infection suggested a switch between innate and adaptive immune responses occurred within the influenza-infected lung between days 5 and 6. To test this hypothesis, we examined the temporal expression of significant TDEGs within the lung for transcripts associated with innate immunity, such as interferon responsive gene set induction, and those associated with adaptive immunity, such as in-migration of activated effector T cells.

We first examined transcripts expressed in the early and delayed expression clusters for those associated with innate or adaptive immune responses (See S3 Table for the full analysis and gene lists). As expected, early transcripts included those of the type I interferon responsive elements and their later downstream associated transcripts, including Irgm1, Ifh7, and Ifih1. Elements associated with the known RIG-I innate viral defense system were also upregulated, including Isg15, Ddx58, Dhx58, and Nfkbib. Other early-activated transcripts were associated with TLR activation and signaling (Stat1, Ccl4, Ccxcl9, Ccxcl0, and Cxcl11).[31, 32] In particular, chemokines to attract inflammatory cells to the lung after infection were rapidly upregulated, including monocyte/macrophage attracting chemokines Ccl2 and Ccl7, and the neutrophil attracting chemokines Cxcl-9, -10, -11, -12, and -15. The lymphocyte attracting chemokines Ccl2 and Ccl17 were prominently upregulated in the early phase as well.

We next examined those clusters with middle and late activation. The lung is the only compartment in which a large proportion (about 30%) of TDEGs show a temporal activation delay. Differences in GO terms between the early and delayed gene modules supported a switch from an innate to an adaptive immune process. Gene ontology functions consistent with an early innate immune response enriched in the early transcriptome clusters included: recruitment of lymphocytes to the lung in response to early infection with chemokines (KEGG pathways mmu04060 and mmu04062), receptor signaling pathways associated with the Type I interferon response (KEGG NOD-like and RIG-I-like pathways); and KEGG mmu04623 cytosolic DNA-sensing pathway which suggests Toll-like receptor expression typical of the early response to influenza.[33] The GO terms enriched in the delayed cluster for the adaptive immune response included T cell proliferation and differentiation (GO:0030217, GO:0042088, GO:0030217, and GO:0042110), of which two terms included genes for distinctive T cell markers such as Cd3, Cd8, and Cd28. Consistent with T cell and B cell receptor modification in naïve or early proliferating lymphocytes, the annotation term adaptive immune response based on somatic recombination of immune receptors built from immunoglobulin superfamily domains (GO:0002460) and the KEGG Jak-STAT signaling pathway [34] annotations appear in the early gene cluster. Given that these transcripts appear in the lung, it is likely that they arise from either mucosal associated lymphoid tissue, or lymphocytes trafficking through the lung from secondary lymphoid organs.

**Fig 3:**
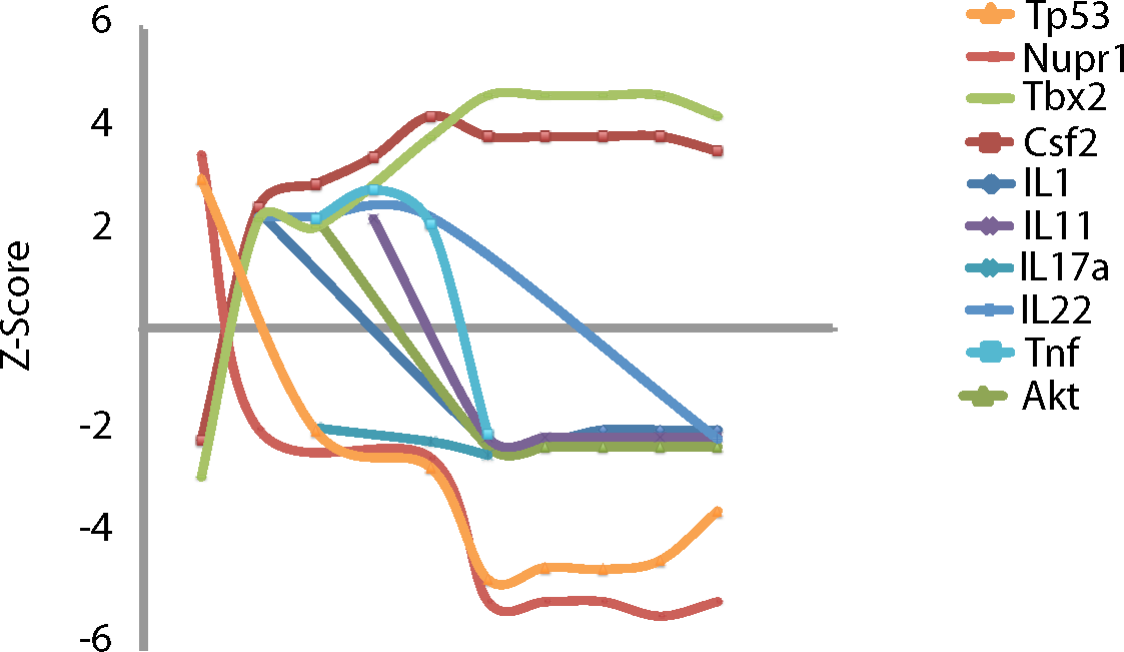
Upstream analysis using delayed TDEGs in the lung compartment. To identify changes in overall gene expression programs, Ingenuity Pathways Analysis (IPA) was used to identify potential upstream modifiers of TDEGs. Z-scores greater than 2.0 indicate probable activation based on downstream gene expression whereas Z-scores less than -2.0 suggest probable inhibition. Scores that switch from positive to negative or the reverse suggest switches in whole gene expression programs or cell types over time. Temporal patterns are evident in upstream modifiers with probable inhibition of TP53 and Nupr1 and concurrent probable activation of Tbx2 and CFS2. Several interleukins and TNF showed initial probable activation and a delayed inhibition. This is consistent with early and mid-infection lung responses including lymphocyte infiltration.

We next examined the delayed gene clusters in lung in more detail, and performed upstream analysis to infer the upstream activated genes that led to the delayed gene module transcription pattern. Within the delayed gene module, several probable upstream modifiers switched from inhibited to activated, or the reverse, between days 1 and 5 (Fig 3). Networks constructed from these probable upstream modifiers (Figs J and K in S1 Text) indicate highly connected nodes that switch from predicted activation to inhibition between days 1 and 2. Among these are several associated with repression of cell growth including tumor promoter Tp53 (p53), nuclear protein 1 (Nupr1), which can complex with Tp53 and mediate cell division, [35] and forkhead box O protein 3 (Foxo3), a member of the FOXO family of transcription factors that are central to control of naïve lymphocyte activation and division [36]. Foxo3 was found as to be itself a TDEG in the delayed cluster. Consistent with these changes, T-box transcription factor 2 (Tbx2), a molecule key to cell cycle control in developing mouse lung [37] was predicted to switch from inhibited to activated during the same period. That these probable upstream modifiers of the delayed gene cluster switch predicted activation states between days 1 and 2 post-infection, provide a plausible link between the early and delayed gene clusters in lung.

### Similar Switch of Innate to Adaptive Responses Seen in Draining Lymph node TDEGs

**Fig 4:**
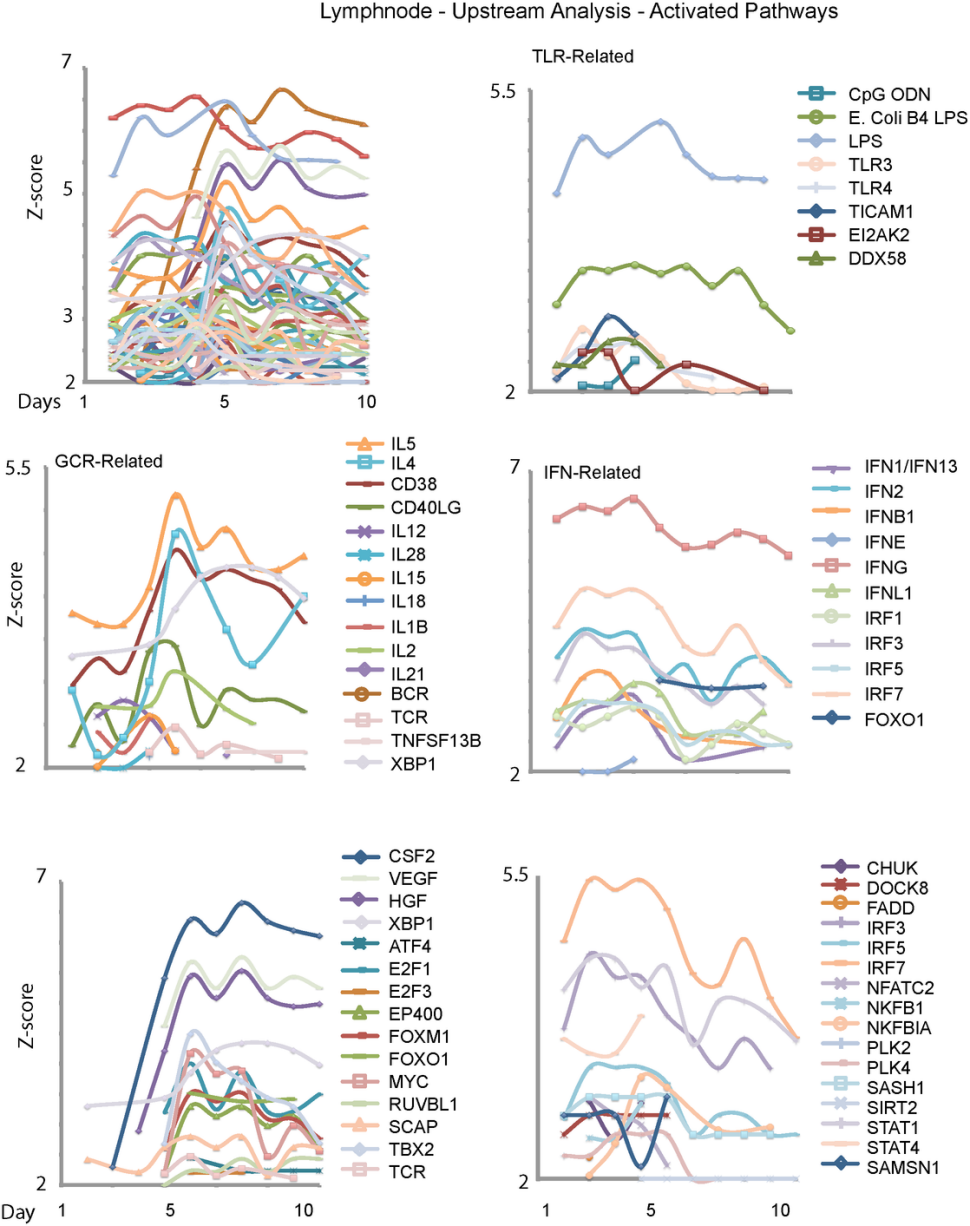
Upstream analysis using all TDEGs in the lymph node compartment. Ingenuity Pathways Analysis (IPA) was used to identify upstream modifiers in lymph node TGED as in Fig 3. (A) The upstream modifiers with probable activation Z-scores. (B) Upstream modifiers related to Toll-like Receptor signaling, consistent with innate-type reactions appeared early in infection. (C) In contrast, upstream modifiers related to the germinal center reaction tended to peak mid-infection. (D) Interferon-related upstream modifiers also tended to peak early in infection. These patterns are consistent with the influx of antigen and initiation of the germinal center response in the draining lymph node. Upstream modifiers associated with growth factors and cell proliferation peaked before Day 5 (D) or after Day 5 (E), suggesting the predominance of different cell types in the lymph node as active germinal center responses evolved.

To examine the switch between innate and adaptive immune responses in the lymph nodes, we examined upstream activators of the statistically significant transcripts. Such analysis allows identification of early signaling pathways associated with the observed downstream transcriptome pattern, and better identifies the transcriptome programs and motifs. In Fig 4, the Z-scores of predicted activated upstream modifiers of lymph node expressed genes were divided into functional groups. Upstream activators that are most significant in days 1 to 5 post-infection were enriched for Toll-like receptor and type I interferon related pathways. Upstream modifiers associated with the adaptive immune response including Tbx2, Foxo1, and the TCR rise in significance after day 5 post-infection and stay elevated to the end of the experiment. These results were consistent with our temporal hypothesis regarding the innate-adaptive immune switch.

While TCR was one upstream modifier identified in lymph node, among the up-regulated TDEGs are a preponderance of B cell-specific genes reflecting the strong wave of B cell expansion. This suggests that the upstream cell cycle control molecules and growth factors identified in common between lung and lymph node may be illuminating common proliferative pathways between T and B lymphocytes responding to influenza infection. Indeed, while the number of delayed genes identified in lymph node was relatively small (n = 45), the associated GO terms were mostly related to cell cycle, cell division, and DNA repair, consistent with proliferation and an active immune response.

### Different Compartments Have Different Periodicity of Temporal Gene Expression Patterns

Next, we clustered genes by the number of modes (local minima/maxima) of their expression patterns over the period of 11 days. This quantity reflects the fluctuations in the expression levels over the period of study. As a special case, a gene is classified as 0-mode if its expression level increases or decreases monotonically over the period of 11 days. The results are summarized in Table 1, which shows that the lung had the least fluctuation (only 1 or 2 modes) while the lymph node has the most fluctuation (1–7 modes). The blood and spleen are intermediate, both having 0–3 modes.

**Table 1:** Number of genes in each mode group.

	0-mode	1-mode	2-mode	3-mode	4-mode	5-mode	6-mode	7-mode
Blood	103	621	104	28	0	0	0	0
Lung	0	1230	412	0	0	0	0	0
LN	0	275	584	594	736	440	223	70
Spleen	89	164	339	22	0	0	0	0

Gene set enrichment analyses show that in lung, mode 2 TDEGs are associated with a few GO terms related to cell cycle/division and regulation of phosphorylation, while mode 1 TDEGs are associated with many more GO terms with diverse functions related to immune responses, such as activation and regulation of many types of cells, several receptor signaling pathways, and various cytokine and chemokine related terms. In the other three compartments, the relationship between modes and immune responses to virus infection are not clear; instead, the overall patterns appear related to different aspects of housekeeping functions in different mode-groups, such as mRNA processing (Mode 0 group) versus DNA processing (Mode 1 group) in spleen. This observation suggests that these different biological processes have different natural periodicities, which are reflected by the number of modes in a fixed time interval.

Based on the smoothed expression patterns, we define a gene as an up-regulated (down-regulated) gene if its estimated maximum mode is larger (smaller) than its baseline (at DPI = 0). All compartments have roughly balanced numbers of up- and down-regulated genes, and GSEA does not show clear functional difference between these two groups.

Finally, we cluster genes based on their activation time which is defined as the time (day) the expression level reaches 50% of its maximum (for up-regulated genes) or minimum (for down-regulated genes). A similar classification was used in [38]. The results are summarized in Table 2. For convenience, we divide the time course roughly into three periods, early (DPI1-2), middle (DPI3-5), and late (DPI6-8). By this definition, most of TDEGs in blood (95.1%) and lymph node (91.4%), as well as the majority of TDEGs in spleen (66.3%), initiate changes in the early phase. In contrast, only 23.6% of the TDEGs in the lung changed during the early phase. The lung was the only compartment with late activation, with 35.0% of lung TDEGs. These observations suggest that most gene regulation events occurring during primary influenza infection are completed in the lymph node, spleen, and blood during the first three days after infection; the delayed activation time for the TDEGs in the lung may be due to the trafficking of large number of immune cells from other compartments to the lung 5–10 days after influenza infection reflecting the switch from the innate to the adaptive immune response.

**Table 2:** Number of genes activated each day.

	DPI-1	DPI-2	DPI-3	DPI-4	DPI-5	DPI-6	DPI-7	DPI-8
Blood	158	656	42	0	0	0	0	0
Lung	0	387	249	164	274	400	117	51
LN	0	2622	68	163	69	0	0	0
Spleen	35	373	184	22	0	0	0	0

### Reconstructing the Gene Regulatory Network

We next used a model based on linear ODE system to construct the compartment specific regulatory network between the clusters induced by influenza infection (refer to Materials and Method section). Briefly, the network structure was determined by a variable selection using the smoothly clipped absolute deviation (SCAD) method [39]. Since the SCAD method relies on appropriately chosen tuning parameters, differences in tuning parameters can affect the reconstructed network. To construct a robust network, we selected edges that were conserved over 50 random perturbations of the tuning parameters and within 20% of their original values (summarized in Table 3). However, these perturbations had very little effect on the original results (Table 3). The trajectories predicted by the model are very close to the smoothed mean curve (blue) of the cluster (Fig 2 and Figs F-H in S1 Text). Thus, the model exhibited consistent and robust behavior within the scope of the tuning parameters.

**Table 3:** Number of Edges in the Linear ODE Networks for each Compartment.

	Clusters	Edges	Network Density	Common Edges (perturbed)
Blood	12	49	0.34	45.24
Lung	32	142	0.14	122.98
LN	24	100	0.17	93.26
Spleen	16	78	0.3	72.38

The last column includes the average number of common edges when perturbing the running parameters.

One notable feature of the reconstructed regulatory networks is the existence of hub clusters that regulate more clusters than most other clusters. We identified clusters C12 (Delay.Up.ActDay8.Modes1), C23 (Early.Up.ActDay2.Modes1), and C30 (Early.Up.ActDay6.Modes2) as the hub clusters for the lung network. These hub clusters have both incoming and outgoing edges, indicating that the genes in the clusters are regulated by, and are regulating, other clusters. This is also evident from their functional enrichment, for example, C23 is enriched for cytokine regulation and processes that lead to the induction of T and B cell activation.

## Discussion

In this manuscript, we report one of the most comprehensive transcriptional analyses to date of the immune response to primary influenza infection. The collection of frequent time-series data from lung, lymph node, spleen, and peripheral blood allowed us to examine, simultaneous, temporal patterns of gene expression after infection within multiple compartments. For each compartment, ODE network models were fit to the data, revealing a systems-level structure of the influenza immune response. Our study shows that at the transcriptome level, each of the four biological compartments respond to influenza infection very differently. These compartmental differences are manifested by vastly different lists of genes with statistically significant changes in expression levels, co-expression modules and their temporal patterns, and the reconstructed GRNs. We believe in depth study of these differences can help researchers design better experiments in the future. For example, we note that the activation and peak up- (down) gene regulation times are different between compartments. The median activation time occurs on DPI 5 for lung and on DPI 2 for the other compartments. However, the median peak/nadir days for these compartments are: DPI 5 (blood and lymph node), 7 (lung), and 9 (spleen).

A related finding is that lymphocytes in the spleen are activated early (DPI 2) but peak transcription levels occur late. This suggests that although spleen responded to influenza infection quickly in the beginning, it takes more than a week for a typical TDEG to reach its largest differential expression (as compared with the base line level). These findings have implications for study design and statistical power. Based on our findings, we suggest that in cross-sectional or a longitudinal studies with limited number of time points, different data collection times should be tailored to the known expression periods of each tissue compartment.

**Fig 5:**
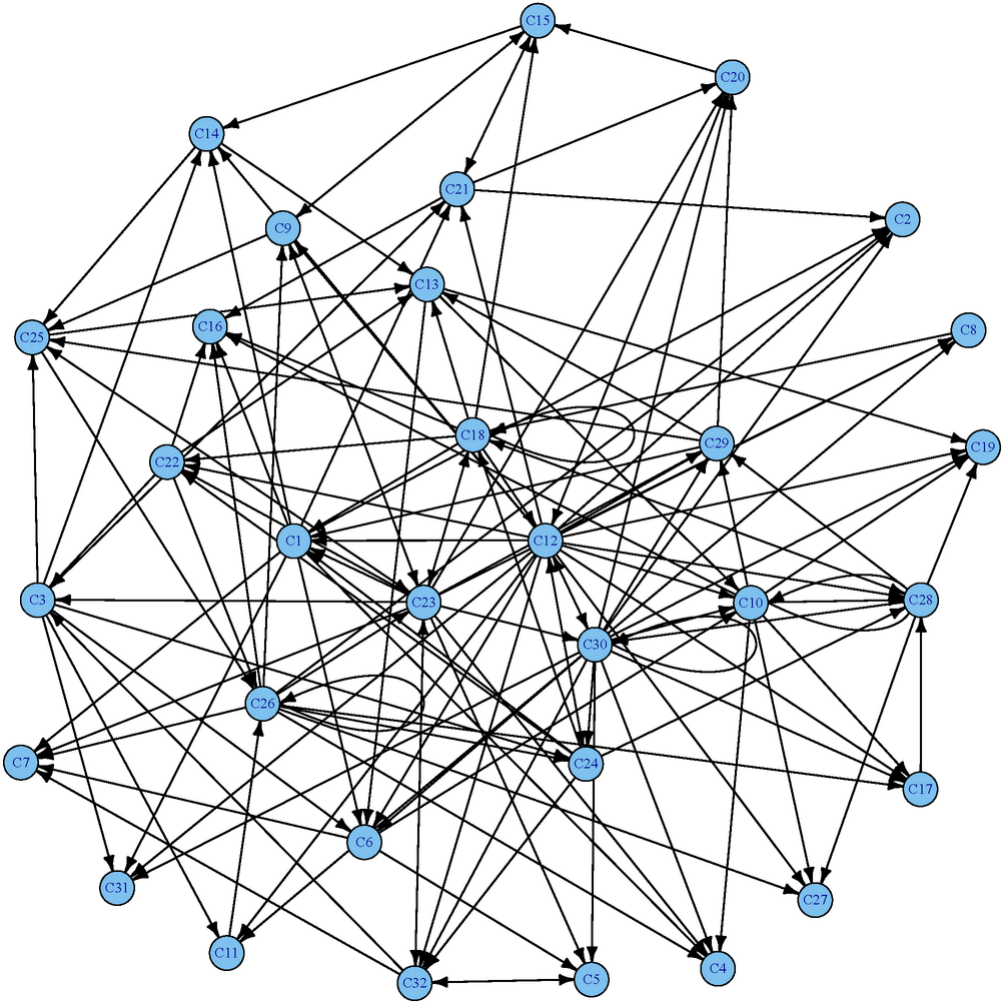
The network of the lung compartment constructed by the linear ODE model. Detailed information about these nodes can be found in Table F of S1 Text.

Our approach also revealed a different GRN within each tissue compartment, which is a new aspect of primary influenza infection. The characteristics of the influenza immune response within each compartment differed by both the number of modules and network density of the GRN. The density of a network is calculated as the ratio of the number of connected edges to the number of all possible edges, allowing for loop (e.g. self-regulatory feedback) edges. A low network density indicates a sparser network. While the lung has the most diverse temporal patterns in terms of the number of clusters, it showed the lowest network density, which suggests that each cluster has fewer average regulatory relationships with others (Fig 5). This finding is consistent with the diversity of cell types involved in the innate and adaptive immune response to influenza infection within the lung, as contrasted with the relatively restricted number of active cell types in the lymph node and spleen. Specifically, the early response induced by lung epithelial cells leads to activation and migration of antigen presenting cells to the lymph node and spleen, with activation of T and B cells in the lymph node and spleen and within the local mucosal immune system. In short, the lung has the most numerous cellular processes, including inflammation, innate and adaptive cellular immune responses, tissue regeneration and repair, and specific anti-viral responses. This likely explains the greater number of network nodes, but sparse network connectivity. Moreover, some clusters such as C11 and C31 have fewer outgoing edges and are typically induced at later time points. These clusters are enriched for adaptive immune response such as T cell receptor signalling, which is consistent with the known trajectories of the influenza immune response. In contrast, the other compartments (blood, lymph node, and spleen) have dense network connectivity with fewer nodes, reflecting more uniformity of gene modules and activated cell types. The blood has the fewest clusters yet they tend to link with each other more tightly. This may be a reflection of the temporal migration of T and B cells at a specific stage of differentiation, with synchronized gene expression, or may alternatively represent continuous communication between different expressed gene clusters (Fig L in S1 Text). As for the lymph node and spleen, which are primarily the sites of T and B cell activation and proliferation, fewer terminal processes are enriched, giving an intermediate transcriptome regulatory network density (Figs M and N in S1 Text).

In summary, we used a systems-biology approach to identify both broad coordination of the influenza immune response across lung, lymph node, spleen and blood, as well as compartment-specific dynamics of gene expression reflecting more focused and specialized biological functions (T and B cell activation and proliferation) within primary lymphoid organs. Broadly expressed genes highlighted for the first time by this analysis included Ddex4, a mediator of the Type I interferon response and RigI signalling pathway, and Ehd4, which may be responsible for increased MHC-I presentation of influenza viral peptides, facilitating CD8 killing of infected epithelial cells. This higher level analysis demonstrates the power of analysing temporal gene expression network topology to gain further insights into complex intra- and cross-compartmental dynamics during infection.

## Materials and Methods

### Experimental Design

Female C57/BL6 mice, 6–12 weeks of age (Jackson Laboratory, Bar Harbor, ME) were anesthetized with intraperitoneal injection of 2,2,2-tribromoethanol (Avertin**™,** Sigma-Aldrich, St. Louis, MO) and inoculated intranasally with 0.03 ml of 1x10^5^ EID_50_ H3N2/Hong Kong/X31 IAV. Mice were monitored for signs of infection throughout the experiment. All animals displayed signs of infection by Day 2 post infection, including ruffled fur, lethargy and initial weight loss. Weight loss peaked around day 6 post infection, with animals typically displaying ~15% weights loss as compared to Day 0 weights. Animals recovered all weight, and exhibited normal behaviour by Day 8 post infection. The temporal trend of body weight loss is illustrated in Fig Q in S1 Text. Only two animals were found dead during the course of the experiment, one on Day 3 and another on Day 9. Both animals were excluded from the study. Mice were sacrificed in groups of six daily for the days post-infection (days 1 to 10) with one set sacrificed before infection (denoted as t = 0-) and one set sacrificed immediately following infection (day 0). Since the animals sacrificed on Day 0- were not infected; these data were used in quality assurance analyses but not mathematical modelling for immune response to virus infection. Serum, peripheral blood mononuclear cells, lung, spleen, and mediastinal lymph nodes tissues were collected daily per group and pooled from two mice in each sample for microarray analysis (12 groups, 3 samples per group, 36 samples, n = 72 mice). For spleen, an additional sample was collected on DPI = 2 so the total sample size was 37. We choose to focus on the first 10 days post infection, as our previous results have demonstrated that the peaks of both the innate and adaptive responses occurs within the first 10 days after infection [8, 40, 41]. All experiments involving animals were reviewed and approved by the Institutional Animal Care and Use Committee (the University of Rochester Committee for Animal Resources).

### Sample Collection and Processing

A small sample of blood was collected by mandibular bleed for serum samples, then the mice were euthanized with a lethal dose of 2,2,2-tribromoethanol and blood was collected by cardiac puncture. The lungs were perfused with phosphate buffered saline (Gibco, Grand Island, NY), removed, and stored on ice until processing. Lungs from two mice in each group were flash-frozen in RNALater buffer (Sigma-Aldrich) and lungs from the remaining three mice were processed. Lung tissue was disrupted by pressing the tissue through a strainer with a syringe piston. The cells were filtered through Nitex mesh (Thermo-Fisher, Waltham, MA) and layered on Lympholyte (Cedarlane, Burlington, NC). TER-119 immunomagnetic beads (BD Bioscience, San Diego, CA) were used to remove red blood cells. For blood samples, 13 were stored in a different buffer, and a subsequent quality assurance microarray analysis of these samples showed a different expression pattern than the remaining 23 samples and thus these 13 samples were excluded from the final blood microarray analysis (Fig O in S1 Text highlights the differences observed between these samples). Lymph nodes and spleen were also collected, dissociated with a dounce homogenizer, lymphocytes isolated by density gradient centrifugation and removal of RBC by TER-119 immunomagnetic beads, and flash frozen in liquid nitrogen. Flow cytometric, ELISpot, and RNA microarray analysis were performed on all cell samples. In addition, ELISA assays were performed on serum samples.

### RNA Isolation and Microarray Analysis

The cells were lysed in RLT buffer (Qiagen, Germantown, MD). The lysates were immediately passed over QiaShredder columns (Qiagen), and flash frozen in liquid nitrogen. Total RNA was reverse transcribed to cDNA with the NuGEN® amplification system, and this cDNA was hybridized with Affymetrix® Mouse Gene 1.0 ST arrays. Quantile-normalized data were generated with Affymetrix® Expression Console software using the PLIER algorithm. Probe sets were mapped to gene symbols with annotations provided by Affymetrix®. All microarray images and processed data are publicly available at the NCBI Gene Expression Omnibus website under accession number GSE57455.

We exclude probe sets with all measurements < 100, a threshold below which it is hard to differentiate gene expression signals from the background noise. This results in 30,184, 25,876, 30,590, and 29,285 probe sets for the lungs, lymph nodes, blood, and spleen, respectively. We apply log_2_ transformation to these expression measurements. Exploratory analyses reveal that the lung data had two distinct groups of array results which correlated exactly with buffer effects. The lungs of the 13 mice displayed by red stars were stored in a different buffer from the other 23 mice, which produced a clear buffer effect as illustrated by Fig O in S1 Text. Based on this observation, we decided to remove these 13 mice from the subsequent analyses. No such artifact was observed from other data sets in the lymph nodes, spleen, and blood. We also selected a few notable inflammatory genes (Ifng, Ifi27l1, Mx1, Ifnb1, Il6, and Cxcl10) and plotted the temporal trend of their expression levels in Fig R in S1 Text. By and large, the expression levels collected from different mice on the same day (post infection) are similar as compared with between-DPI or between-gene variation.

### Differential Expression Analysis

For each of the four compartments, we want to identify genes whose time course gene expressions have significant changes from the baseline. The null hypothesis of this testing problem can be defined as:

$$H_{0}:{\text{x}}_{i}(t)=x_{i}(t),\;0\leq t\leq 10,\;i=1,2,\ldots ,n,$$ (1.1)

where *x*_*i*_(*t*) represents the underlying true expression curve for the *i*th gene. The baseline is chosen to be the gene expression level on DPI = 0, since the mice on DPI = 0 were killed immediately after receiving flu virus and it is reasonable to assume that the gene expression levels had not been affected. In practice, the true expression curve *x*_*i*_(*t*) needs to be estimated from the noisy microarray data, *y*_*ijk*_ = *x*_*i*_(*t*_*j*_)+ *ϵ*_*ijk*_, where *j* = 0,…,10 indexes the time points and *k* indexes the repetitions at each time point. A commonly used smoothing technique is to expand *x*_*i*_(*t*) in terms of an intercept plus an *L*-dimensional linear basis:

$$x_{i}(t)=c_{i0}+\overset{L}{\underset{l=1}{\sum }}c_{il}\eta_{l}(t),$$ (1.2)

In this study the basis functions *η*_*l*_(*t*), *l* = 1,2,…,*L*, are eigen-basis estimated from the data by the functional principal component analysis (FPCA) [42]. These orthogonal basis functions reflect the major modes of variation in the data, and often fewer basis functions are needed to capture the shape of the gene expression trajectory compared to that using fixed basis functions. Analogous to the *t*- and *F*-statistics, we use a test statistic that compares the goodness of fit of the model under the null hypothesis to that under the alternative hypothesis [20]:

$$F_{i}=\frac{SS_{i}^{0}-SS_{i}^{1}}{SS_{i}^{1}},$$ (1.3)

where $SS_{i}^{0}$ and $SS_{i}^{1}$ are the sums of squared residuals obtained from the model fits under the null and alternative hypotheses, respectively. A permutation method is used to compute the *p*-values, in which each permutation sample $y_{ij*k}^{P}$ is *y*_*ijk*_ with the index *j* randomly permuted.

With the null statistics $F_{i}^{r}$ calculated from *R* permutation samples, we can then compute the unadjusted *p*-value for each gene:

$$p_{i}=\overset{R}{\underset{r=1}{\sum }}\frac{\#\{j:F_{j}^{r}\geq F_{i},\;j=1,\ldots ,n\}}{n\cdot R}.$$ (1.4)

The multiple test correction method proposed by [43] is then applied to control the false discovery rate (FDR). See [20] for more details about the methodology. The top two leading eigen-functions of all compartments are illustrated in Fig P in S1 Text.

### Cluster Analysis

We cluster the differentially expressed probe sets into different groups or functional modules based on their time course expression patterns by considering four features hierarchically: 1) whether there is a time delay for the probe set to start express, compared to the baseline; 2) the number of modes (local minima or maxima) of the expression patterns from Day 0 to Day 10; 3) whether the probe set is up or down regulated; and 4) the activation time which is defined as the time to reach 50% of the first peak or nadir (minima or maxima). Since the analysis of the time course expression patterns may be sensitive to excessive variation of one time point or outliers, it is necessary to remove these outliers before clustering analysis. We ran a residual analysis to identify these outliers. We observed that DPI = 1 is associated with abnormally high RSS for the lung and lymph node data, thus we removed DPI = 1 and refit the lung and lymph node data before the clustering analysis. For each significant probe set, we first average the expression values on the same day and standardize the data by the mean and standard deviation over time. The following clustering method is applied to these standardized data so that the grouping of probe sets is based on the gene expression pattern rather than the expression magnitude. After exploring the gene expression patterns visually, we found that some of the probe set expressions do not change from baseline for the first several days after influenza infection. Thus, we first clustered probe sets into two big classes: whether there is a time delay for the probe set to start express, compared to the baseline, which we call them as delay probe sets and early probe sets. We only consider the delay window from Day 3 to Day 5 based on our preliminary data exploration. A linear regression model for the expression data for each probe set for the first 3, 4, or 5 days can be written as:

$$y_{it}-y_{i0}=a_{im}t+\epsilon_{it},\;t=0,2,\ldots ,m,\;m=3,4,5.$$ (1.5)

Here *y*_*it*_ is the average expression of the *i*th gene on the *t*th day; *m* is the upper limit of delayed time (day). If there are *m*-day delay for the *i*th gene's expression, we expect that *a*_*im*_ = 0; consequently we may use a standard *t*-statistic to test *a*_*im*_ = 0 and identify the delayed genes. At the same time, we also need to exclude genes with very large ${\hat{a}}_{im}$ (the estimated *a*_*im*_) or very large standard error of ${\hat{a}}_{im}$. Based on the above reasoning, we define delayed genes by the following criteria:

1. Either the *t*-statistic is less than 3.0 or ${\hat{a}}_{im}<0.15$; and
2. The adjusted standard error of ${\hat{a}}_{im}$, $s_{im}≔\sqrt{m-1}\hat{\sigma }({\hat{a}}_{im})$, is less than 0.12.

These thresholds are determined based on our visual inspection of the gene expression patterns. Next, we cluster genes by the number of modes (local minima/maxima) of their expression patterns over the period of 10 days. The following procedure is used for this purpose.

1. Smooth the expression data by the FPCA technique describe in Section “Differential expression analysis”.
2. An interior time point (*j* = 2,3,…,9) is called a *mode* if for a given tolerance level *δ* = 0.1, either one of the following criteria is met:

$$\{\begin{array}{ll} \hat{g}(t_{i})-\hat{g}(t_{i-1})>\delta \;\text{and}\;\hat{g}(t_{i})-\hat{g}(t_{i+1})>\delta ; & \text{the}\;"\text{V}"\;\text{case}\\ \hat{g}(t_{i})-\hat{g}(t_{i-1})<-\delta \;\text{and}\;\hat{g}(t_{i})-\hat{g}(t_{i+1})<-\delta ; & \text{the}\;"\Lambda "\;\text{case} \end{array}$$ (1.6)

Here *x*_*ij*_ is the smoothed average expression level of the *i*th gene on the *j*th DPI. If the number of genes in a mode-group is too small (*n* < 50), it is merged to a neighboring group. For example, there are only 18 genes in the group of 0-mode for the lung data, thus it is merged with the group with 1-mode. Based on the smoothed expression patterns, we define a gene as an up-regulated (down-regulated) gene if its most prominent mode is larger (smaller) than its baseline (at DPI = 0). Finally, we cluster genes based on their activation time which is defined as the time (day) the expression level of a gene reaches 50% of its maximum (for up-regulated genes) or minimum (for down-regulated genes).

After classifying the TDEGs into unique clusters based on the above criteria, we noticed that some clusters have only a handful of genes, so we merge small clusters (with n ≤10 genes) to a large cluster most similar to it in terms of the shape of the mean curves.

### Reconstructing the Gene Regulatory Network

We adopt the following model based on linear ODEs proposed by [44] to describe the dynamic gene regulatory network (GRN):

$$\{\begin{array}{cc} \frac{\text{d}X_{i}(t)}{\text{d}t}=\alpha_{i0}+\sum_{j=1}^{p}\alpha_{ij}X_{j}(t), & \text{for}\;t\in (0,10],\\ X_{i}(0)=X_{i0}, & \text{for}\;t=0. \end{array},\;i=1,2,\ldots ,p,$$ (1.7)

where *X*_*i*_(0) represents the mean gene expression function of the *i*th gene and *X*_*i*0_ represents its initial value. A mixed-effect model is used to account for the between-gene variation within each cluster. The parameter *α*_*i*0_ is the intercept term and *α*_*ij*_ quantifies the regulation effect between clusters in the network. Once genes are properly classified into clusters (modules), we can build an ODE network system for *M*_*k*_(*t*), the mean expression curve of the *k*th cluster (module). In this way, the dimension of Eq (1.7) is greatly reduced, and we have the following system instead:

$$M{'}_{k}(t)=b_{k0}+\overset{K}{\underset{i=1}{\sum }}b_{ki}M_{i}(t),\;k=1,2,\ldots ,K.$$ (1.8)

When *b*_*ki*_ is nonzero, we assign a directed edge between the *i*th and *k*th modules. Since biological systems are seldom fully connected and most modules are only directly connected to a small number of other modules [45], it is commonly assumed that the GRN is a sparse network. We adopt the two-stage smoothing based method [46, 47] for estimating the ODE system (1.8). This approach avoids numerically solving the differential equations and allows independent model selection and parameter estimation for one equation at a time, which significantly reduce the computational cost [44]. We first obtain the estimates of *M*_*k*_(*t*) and their derivatives *M*'_*k*_(*t*) from the observed data. We then substitute these estimates into Eq (1.8) to turn it into *K* independent pseudo regression models. The smoothly clipped absolute deviation (SCAD) [39] is then applied to these pseudo regression models to determine the nonzero coefficients. To overcome the estimation deficiency of the two-stage method, we refine the parameter estimates for the selected ODE model using the nonlinear least squares (NLS) method [48].

To obtain the gene-specific regulatory parameter estimates, we consider the following mixed-effects ODE model [44] for the *k*th module (*k* = 1,2,…,*K*)

$$\frac{dX_{i}(t)}{dt}=\beta_{k0}+\sum_{j\in S_{k}}\alpha_{ij}M_{j}(t)=\beta_{k0}+\sum_{j\in S_{k}}\left(\beta_{kj}+\gamma_{ij})M_{j}(t\right),$$ (1.9)

where *X*_*i*_(*t*) is the true expression curve of the *i*th gene in this module; $S_{k}=\{1\leq i\leq K:\;{\hat{\beta }}_{ki}^{S}\neq 0\}$ is the collection of nonzero coefficients for the *k*-th differential equation; the random effects *γ*_*ij*_ are assumed to follow normal distributions and they characterize the between-gene variation in the *k*th module.

### Functional Annotation and Enrichment Analyses

The probe sets are mapped to gene symbols with annotations provided by Affymetrix®. We first performed the functional enrichment analysis using DAVID [49], through which the GO (gene ontology) annotations [47] and KEGG [50], BioCarta [51], and Reactome [52] pathways enriched in each cluster can be identified. Each functional term was evaluated by its statistical significance test based on the EASE score probabilities, which is more conservative than the standard Fisher's exact test. The significant GO terms and enriched pathways were selected based on a pre-specified threshold (FDR<0.05).

The GO annotations are not specifically designed for immunology related enrichment analyses. In order to decipher more detailed immunology specific functional implications underlying these gene clusters and the regulatory networks, we define 406 key words based on the curated terms listed in Imm-Port (http://immport.niaid.nih.gov) that are related to immunology, such as “B cell", “T cell”, “lymphocyte", etc. We encode these key words into Immunology Ontologies (IO) and annotate each gene by matching the key words with the gene's definition and description in GenBank (http://www.ncbi.nlm.nih.gov/genban). The statistical significance of these keyword related annotations are calculated by the Fisher’s exact test.

Using both functional enrichment from standard annotations and self-defined immunology related functional terms of each cluster; we build a functional landscape of the regulatory network after viral infection by connecting the clusters with the reconstructed gene regulatory network. The enriched annotations indicate the functions and pathways related to immunology are triggered after infection. Moreover, the regulatory linkages provided temporal patterns of the functional implications in the network.

### Upstream Modifier Analysis

Data sets containing TDEG expression values were uploaded into Ingenuity Pathways Analysis (IPA) (http://ingenuity.com/). Each identifier was mapped to its corresponding object in Ingenuity's Knowledge Base. Network eligible molecules were overlaid onto a global molecular network developed and networks were then algorithmically generated based on their connectivity. Upstream analysis modulator Z-scores of 2 and above were considered probable activators and less than −2 were considered probable inhibitors. Excel (Microsoft, Redmond, WA), Adobe Illustrator and Acrobat Professional CS5 (Adobe, San Jose, CA) were used to create Figs 3 and 4.

## Supporting Information

S1 Table This file contains known biological functions of the 11 common TDEGs identified in all four compartments. (XLSX)

S2 Table This file contains results of feature-based gene set enrichment analyses as applied to the blood compartment. (XLSX)

S3 Table This file contains results of feature-based gene set enrichment analyses as applied to the lung compartment. (XLSX)

S4 Table This file contains results of feature-based gene set enrichment analyses as applied to the lymph node compartment. (XLSX)

S5 Table This file contains results of feature-based gene set enrichment analyses as applied to the spleen compartment. (XLSX)

S1 Text This file contains additional results such as the signal-to-noise ratio in each compartment, temporal patterns of common TDEGs in each compartment, etc. (PDF)
