# Supplementary material for: Diversity in Compartmental Dynamics of Gene Regulatory Networks: The Immune Response in Primary Influenza A Infection in Mice
Source: PLoS One. 2015 Sep 28;10(9):e0138110. doi: 10.1371/journal.pone.0138110 (PMC4586376; doi:10.1371/journal.pone.0138110)
Supplement: S1 Text — (PDF) [file pone.0138110.s006.pdf]

Supplementary Text S1 for Diversity in Compartmental Dynamics of Gene Regulatory Networks: The Immune Response in Primary Influenza A Infection in Mice

Xing Qiu[1¶], Shuang Wu[1¶], Shannon P. Hilchey[2], Juilee Thakar[1,3], Zhi-ping Liu[1,4], Stephen L. Welle[5], Alicia D. Henn[2,3], Hulin Wu[1*], Martin S. Zand[2*]

1 Department of Biostatistics and Computational Biology, University of Rochester, 601 Elmwood Avenue, Box 630, Rochester, New York 14642, U.S.A.
2 Department of Medicine, University of Rochester, 601 Elmwood Avenue, Box 630, Rochester, New York 14642, U.S.A.
3 Department of Microbiology and Immunology, University of Rochester, 601 Elmwood Avenue, Box 630, Rochester, New York 14642, U.S.A.
4 Department of Biomedical Engineering, Shandong University, Jinan, Shandong, China.
5 Functional Genomics Center, University of Rochester, Rochester, New York, U.S.A.

Signal-to-noise ratio of gene expressions in each compartment

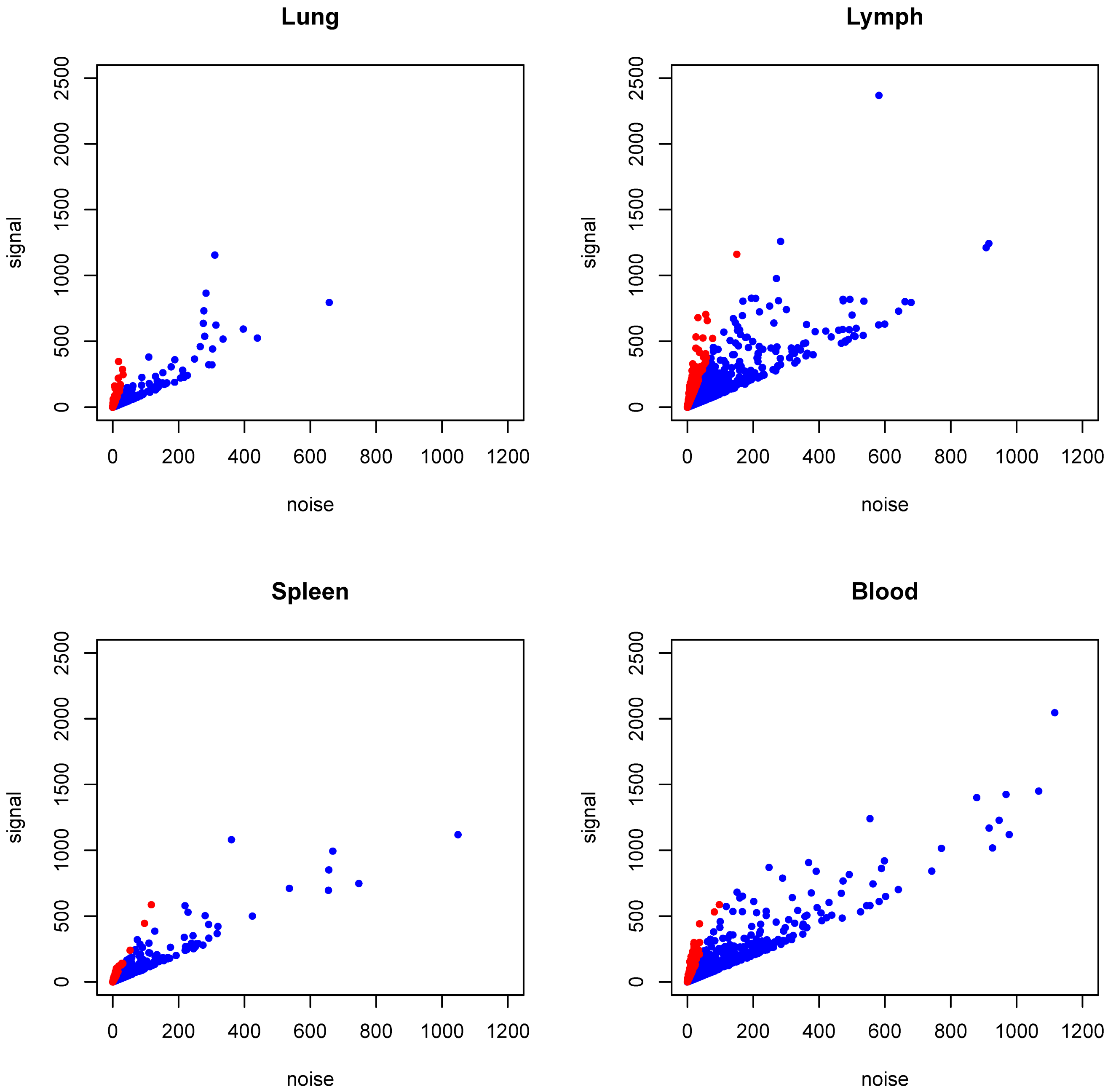

Figure A: **Signal-to-noise ratio of data in each compartment.**

One noticeable fact is the dramatic difference of the number of significant probe sets identified from four compartments (1,906 in lung; 3,548 in lymph node; 224 in blood; and 508 in spleen). Figure A shows the scatterplots of signals versus noises in these data. For each gene (a dot in this figure), its signal is quantified as the L^2-norm of the fitted curve in functional principal component analysis and its noise is defined as the square-root of the residual sums of squares. This figures shows that the blood and spleen data have more noise than the other two compartments and the spleen data have the weakest signals. So we identified fewer significant probe sets for the blood and spleen data. The lymph nodes have largest signal-to-noise ratio and

therefore the largest number of probe sets.

Functional Annotations of common TDEGs

A total of 11 genes are are significant in all compartments: Ddx60, Ehd4, Gm1966, Gm6545, Gm7609, Gvin1, Ly6a, Ly6c2, Ms4a4c, Phf11 and Xaf1. Among them, Gm1966, Gm6545 and Gm7609, are pseudogenes for which functions are not yet clear. The functional annotations of other eight genes are listed in a separate file `Stab1-common-TDEGs`.

Temporal patterns of common TDEGs

Figures B-E shows the temporal patterns of these common TDEGs in four compartments.

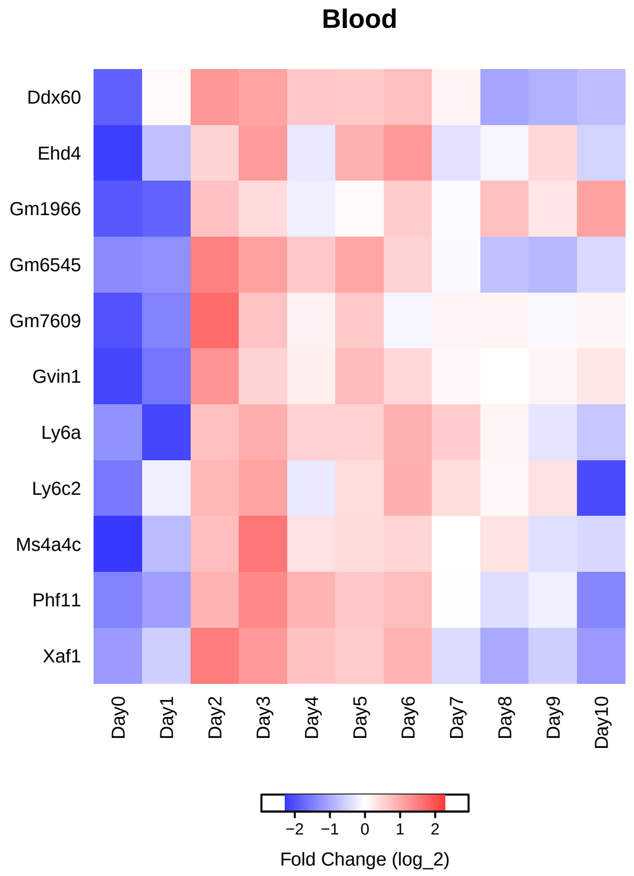

Figure B: **Temporal patterns of 11 common TDEGs in the blood compartment.**

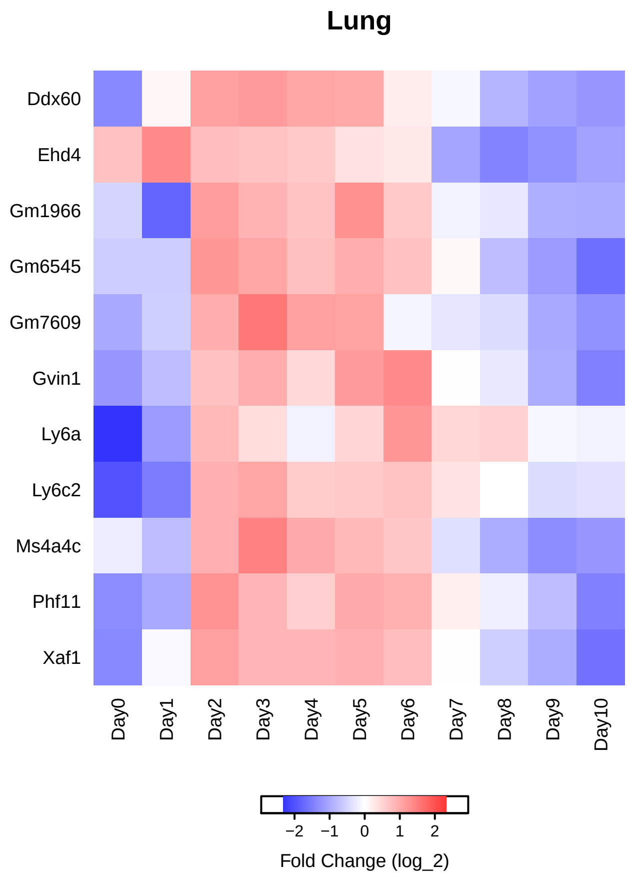

Figure C: **Temporal patterns of 11 common TDEGs in the lung compartment.**

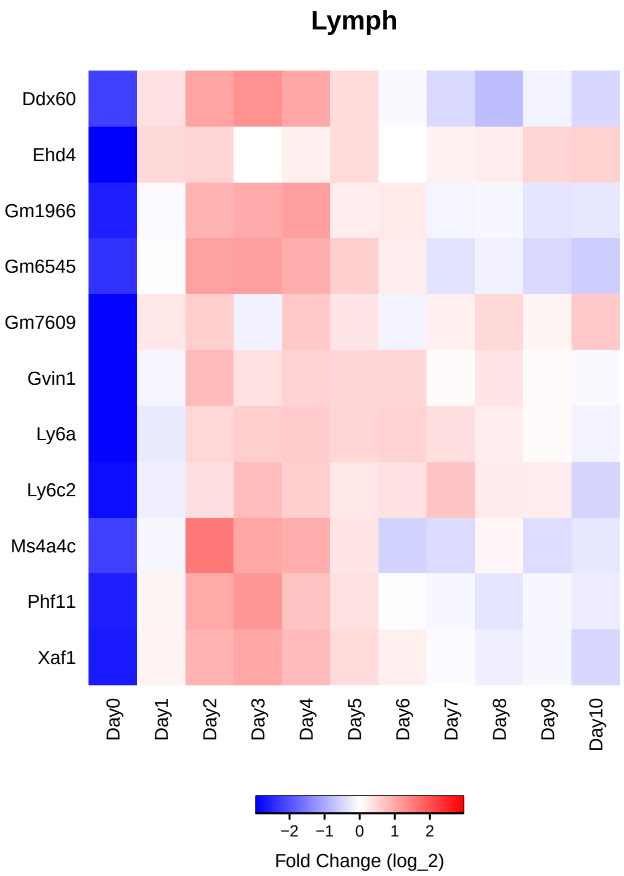

Figure D: **Temporal patterns of 11 common TDEGs in the lymph node compartment.**

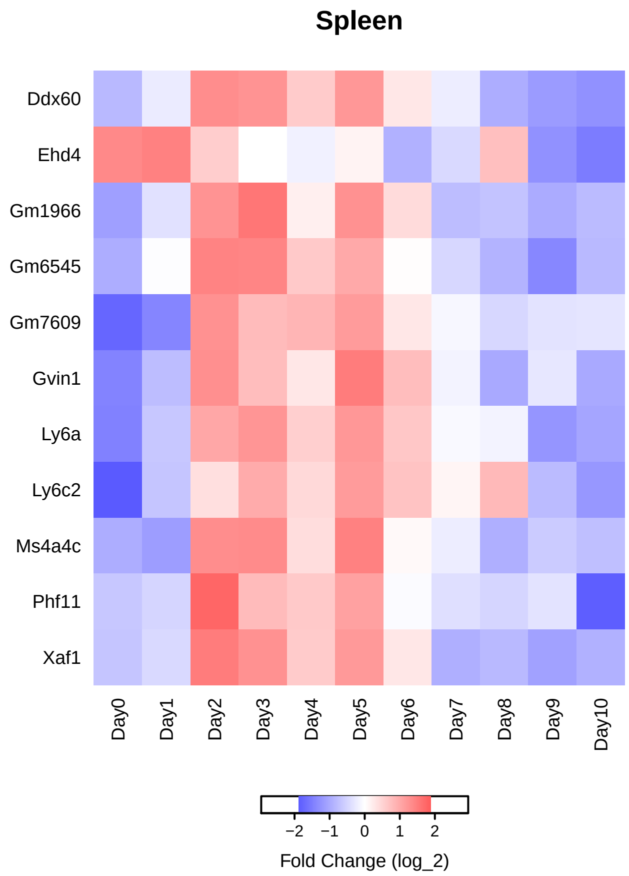

Figure E: **Temporal patterns of 11 common TDEGs in the spleen compartment.**

Line plots

Figures F-H shows The line plots of each cluster in the blood, lymph node, and spleen compartments. Figure 2 in the main manuscript shows the same line plot for the lung compartment.

TDEGs identified in each compartment are classified into co-expression modules. Grey curves represent the temporal trajectory of expression levels for each gene, standardized to zero mean and unit standard deviation. Blue curves are the smoothed mean expression levels for each cluster. Red curves are the predicted mean expression levels from the ODE model. Each co-expression module is classified by four criteria: Delay or Regular; Up or Down; the activation day of its mean gene expression; and number of modes of its mean expression. This information is shown in the subtitles, together with the numbers of genes contained in these modules.

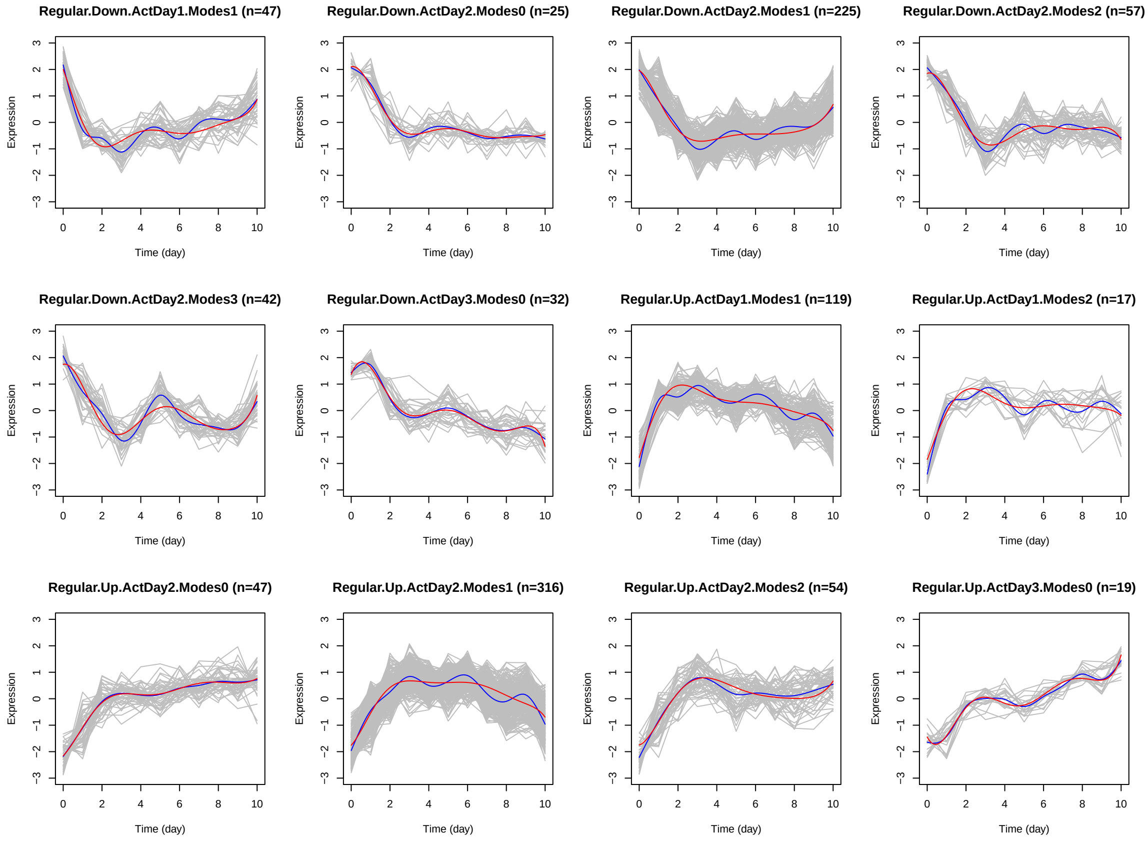

Figure F: **The line plots of each cluster in the blood compartment.**

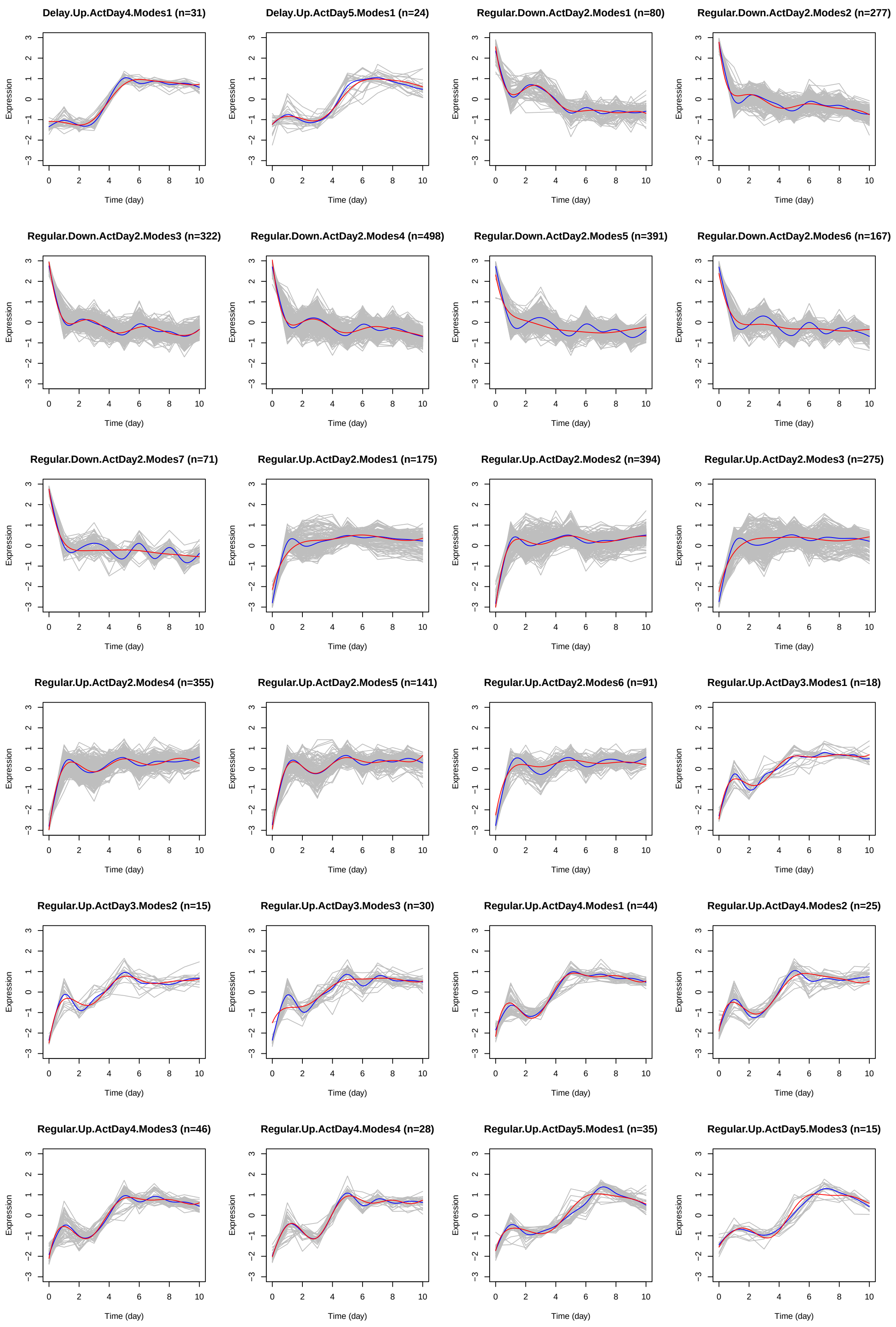

Figure G: **The line plots of each cluster in the lymph node compartment.**

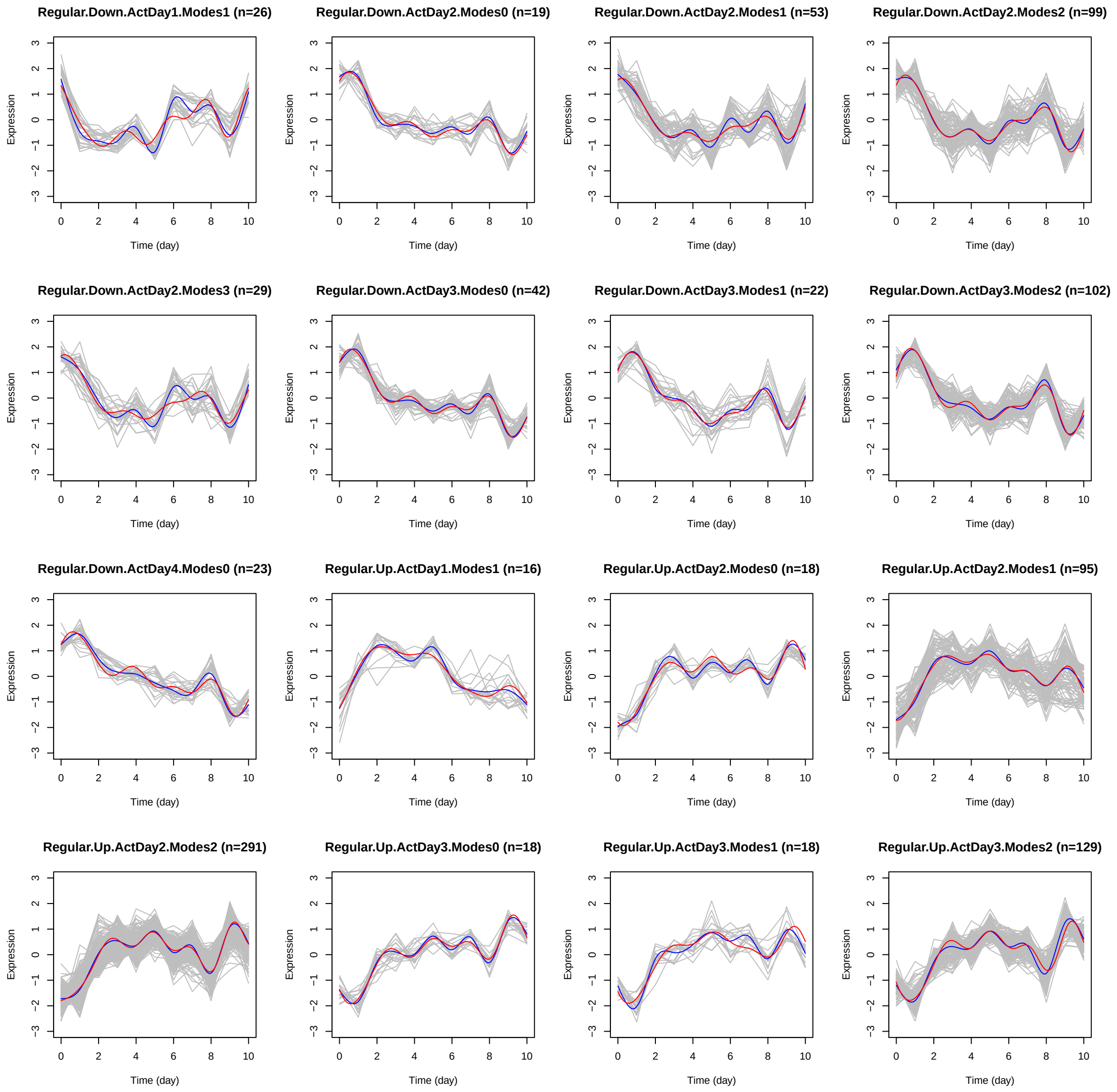

Figure H: **The line plots of each cluster in the spleen compartment.**

Examples of delayed and non-delay genes

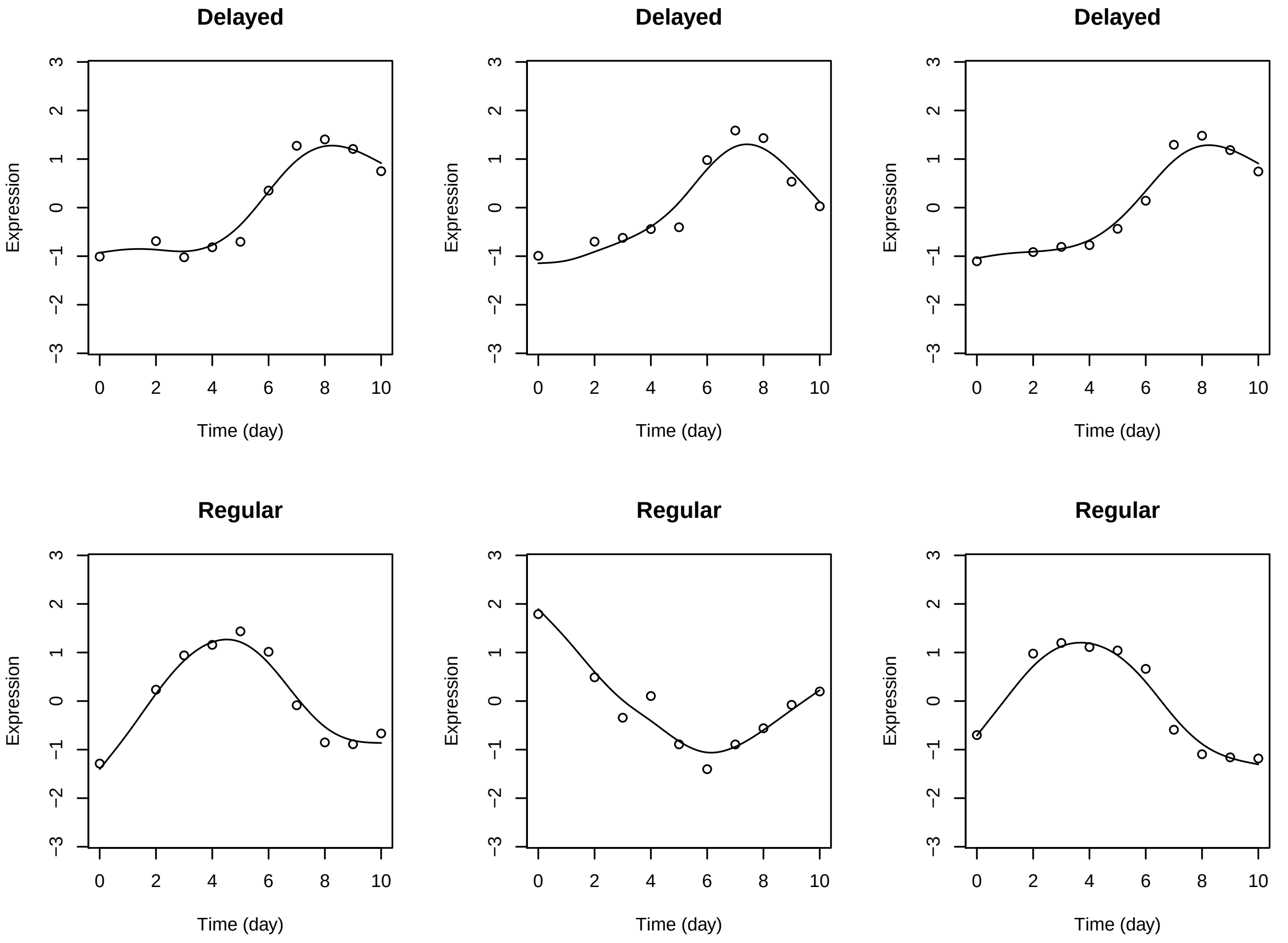

Figure I: **Examples of temporal patterns of genes with or without delay.**

Upstream Analysis

Day 01 Network Day 01 Expression Levels

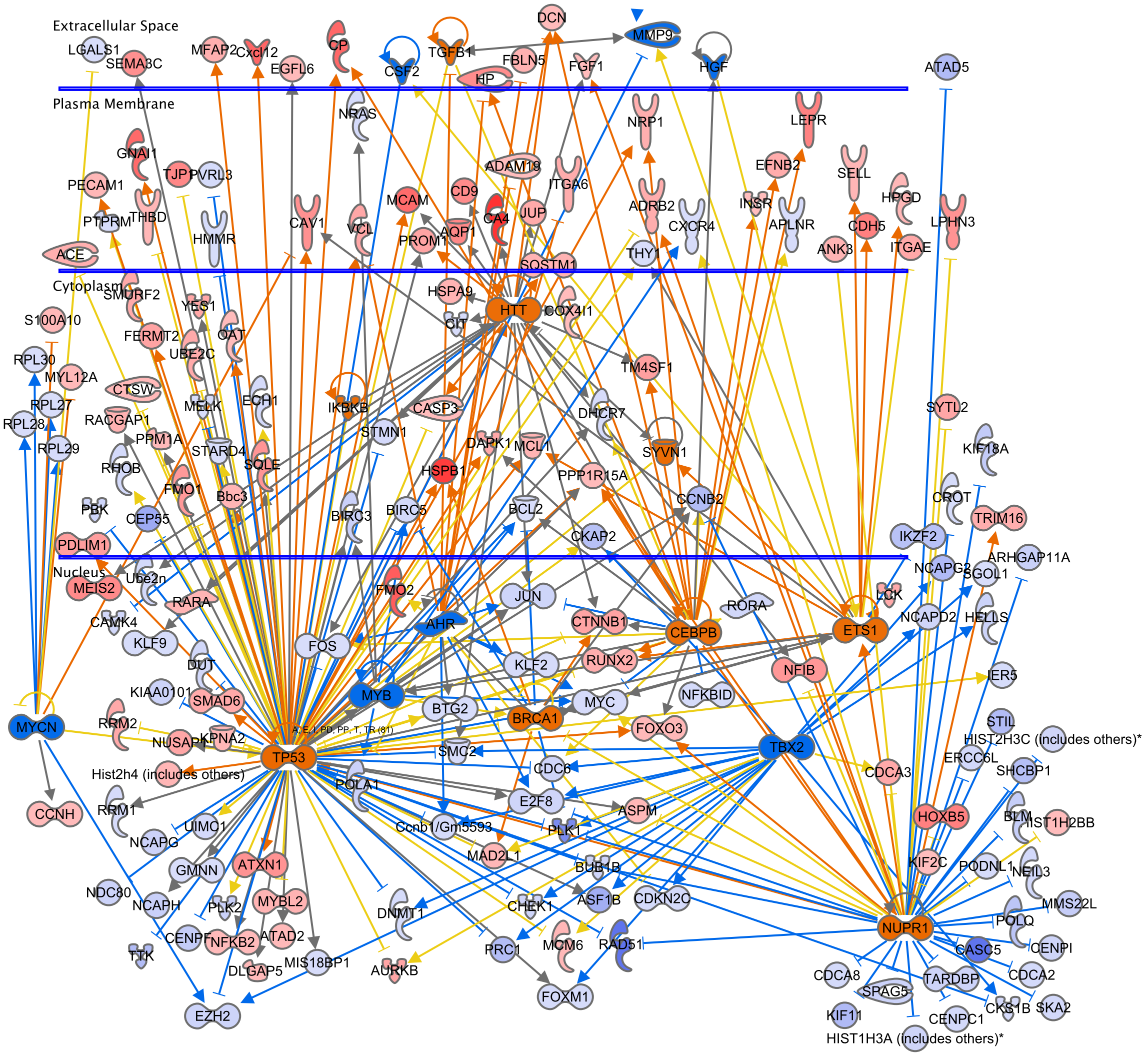

Figure J: **Network built from the delayed TDEGs in lung on DPI 1.**

Day 01 Network Day 02 Expression Levels

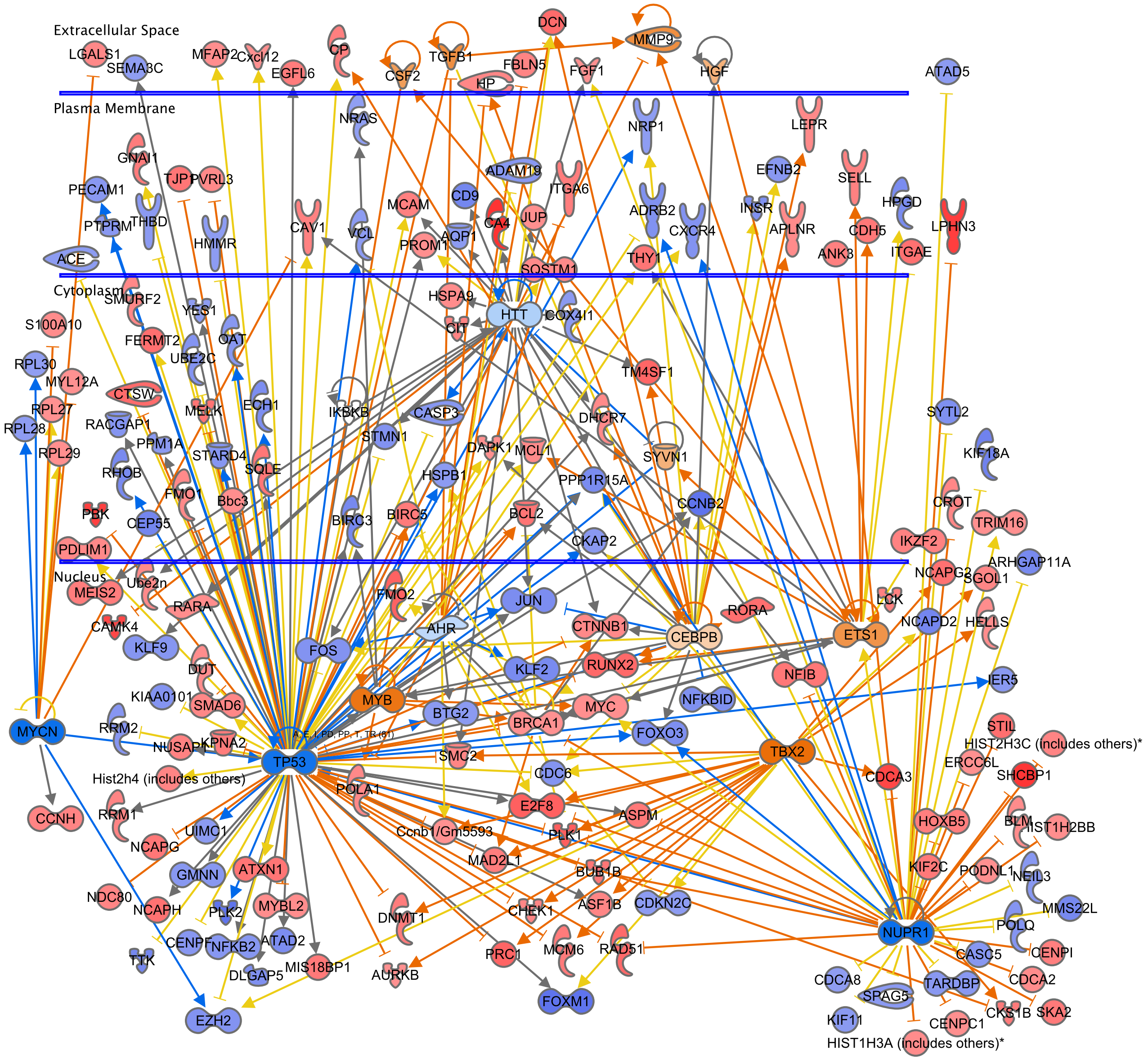

Figure K: **Network built from the delayed TDEGs in lung on DPI 2.**

Feature Based Functional Enrichment Analysis

We conduct functional enrichment analysis for each compartment based on four features: 1. Delay/regular; 2. Number of modes; 3. Activation day; 4. Up/down regulation. These results are provided as separate files

- Blood compartment: `STab2.Blood_Feature_Cluster_Fun`
- Lung compartment: `STab3.Lung_Feature_Cluster_Fun`
- Lymph node compartment: `STab4.Lymph_Feature_Cluster_Fun`
- Spleen compartment: `STab5.Spleen_Feature_Cluster_Fun`

Technical details about this feature based cluster analysis can be found in the "Materials and Methods" section of the manuscript.

Inward and Outward Connections of the Reconstructed Networks

Tables F-I listed the inward and outward connections of the reconstructed gene regulatory networks. Negative signs in these tables indicate negative coefficients in the ODE model. Figure 9 in the manuscript (network reconstructed from the lung compartment) and Figures L-N are the graphical representations of these networks.

	ID	Cluster.Name	Inward	Outward
1	C1	Delay.Down.ActDay4.Modes1	12-,18-,23-,24-,29-	2-,4-,6-,7,14,16-,21-,31
2	C2	Delay.Down.ActDay5.Modes1	1-,12-,21-,26-,30-	
3	C3	Delay.Down.ActDay5.Modes2	21-,22,23-,32-	6,11,14-,21,24,25,31
4	C4	Delay.Down.ActDay6.Modes1	1-,10,23-,26-,30-	
5	C5	Delay.Down.ActDay6.Modes2	6-,23-,30-,32-	32-
6	C6	Delay.Down.ActDay7.Modes2	1-,3,12,13,30-	5-,7,10-
7	C7	Delay.Up.ActDay5.Modes1	1,6,23-,26,32-	
8	C8	Delay.Up.ActDay6.Modes1	12-,23-,30	18-
9	C9	Delay.Up.ActDay6.Modes2	12-,15,23,26	15-,23-,25-
10	C10	Delay.Up.ActDay7.Modes1	6-,10-,12,13-,30	4,10-,16-,17,19,27-,28-,29-
11	C11	Delay.Up.ActDay7.Modes2	3,12-,30	26-
12	C12	Delay.Up.ActDay8.Modes1	18-,20,24-,30	1-,2-,6,8-,9-,10,11-,14-,17-,19-,21,22,24,27,28,29,30-,31-,32-
13	C13	Regular.Down.ActDay2.Modes1	14-,18-,22-,25-,29	6,10-,19-
14	C14	Regular.Down.ActDay2.Modes2	1,3-,12-,15	13-,25
15	C15	Regular.Down.ActDay3.Modes1	9-,18-,20,21-	9,14,21
16	C16	Regular.Down.ActDay4.Modes1	1-,10-,18,21-,22,26-	
17	C17	Regular.Down.ActDay5.Modes1	10,12-,26-,30-	28-
18	C18	Regular.Down.ActDay5.Modes2	8-,18-,23-,24-,28	1-,12-,13-,15-,16,18-,22,23,28-
19	C19	Regular.Down.ActDay6.Modes1	10,12-,13-,28-,30-	
20	C20	Regular.Down.ActDay6.Modes2	21-,23-,29,30-	12,15
21	C21	Regular.Down.ActDay7.Modes2	1-,3,12,15	2-,3-,15-,16-,20-
22	C22	Regular.Down.ActDay8.Modes1	12,18,23,24-	3,13-,16,26-
23	C23	Regular.Up.ActDay2.Modes1	9-,18,25-,26,32-	1-,3-,4-,5-,7-,8-,9,18-,20-,22,25,29-,30-,32-
24	C24	Regular.Up.ActDay2.Modes2	3,12,26,30-	1-,12-,18-,22-,28,32-
25	C25	Regular.Up.ActDay3.Modes1	3,9-,14,23,29	13-,23-,26-
26	C26	Regular.Up.ActDay4.Modes1	11-,22-,25-,26	2-,4-,7,9,16-,17-,23,24,26,27
27	C27	Regular.Up.ActDay5.Modes1	10-,12,26,28	
28	C28	Regular.Up.ActDay5.Modes2	10-,12,17-,18-,24	18,19-,27,29,30-
29	C29	Regular.Up.ActDay6.Modes1	10-,12,23-,28,30	1-,13,20,25
30	C30	Regular.Up.ActDay6.Modes2	12-,23-,28-,30	2-,4-,5-,6-,8,10,11,12,17-,19-,20-,24-,29,30,31,32-
31	C31	Regular.Up.ActDay7.Modes2	1,3,12-,30	
32	C32	Regular.Up.ActDay8.Modes1	5-,12-,23-,24-,30-	3-,5-,7-,23-

Table F: Inward and outward connections of the reconstructed gene regulatory networks for the **lung** compartment. Negative signs in these tables indicate negative coefficients in the ODE model.

	ID	Cluster.Name	Inward	Outward
1	C1	Regular.Down.ActDay1.Modes1	5, 10, 11, 15-, 16	2, 3, 4, 5-, 6, 8, 9, 10-, 11-, 12-, 13-, 14-, 15-, 16-
2	C2	Regular.Down.ActDay2.Modes0	1, 2, 11, 16	2, 4, 6, 7, 8, 9, 11, 12-, 14-, 15, 16-
3	C3	Regular.Down.ActDay2.Modes1	1, 3, 10, 11, 16	3, 5
4	C4	Regular.Down.ActDay2.Modes2	1, 2, 10, 11-, 16	11
5	C5	Regular.Down.ActDay2.Modes3	1-, 3, 5, 16	1, 5, 6-, 7, 9-, 14
6	C6	Regular.Down.ActDay3.Modes0	1, 2, 5-, 11, 16	
7	C7	Regular.Up.ActDay1.Modes1	2, 5, 10-, 11, 16	9
8	C8	Regular.Up.ActDay1.Modes2	1, 2, 11, 15, 16	
9	C9	Regular.Up.ActDay2.Modes0	1, 2, 5-, 7, 16	10, 13
10	C10	Regular.Up.ActDay2.Modes1	1-, 9, 10-, 11, 12-	1, 3, 4, 7-, 10-, 11-, 12-, 15-
11	C11	Regular.Up.ActDay2.Modes2	1-, 2, 4, 10-, 11	1, 2, 3, 4-, 6, 7, 8, 10, 11, 12-, 13-, 14-, 15, 16-
12	C12	Regular.Up.ActDay3.Modes0	1-, 2-, 10-, 11-, 16-	10-

Table G: Inward and outward connections of the reconstructed gene regulatory networks for the **blood** compartment. Negative signs in these tables indicate negative coefficients in the ODE model.

	ID	Cluster.Name	Inward	Outward
1	C1	Delay.Up.ActDay4.Modes1	1, 2-, 15-, 18	1, 2, 4-, 11-, 12-, 13-, 15, 17-, 20, 23
2	C2	Delay.Up.ActDay5.Modes1	1, 9, 15, 18, 24-	1-, 6, 9-, 18-, 20-, 22
3	C3	Regular.Down.ActDay2.Modes1	10, 15, 18-, 20-, 24	
4	C4	Regular.Down.ActDay2.Modes2	1-, 15, 20, 23	24
5	C5	Regular.Down.ActDay2.Modes3	7, 8-, 10, 20	
6	C6	Regular.Down.ActDay2.Modes4	2, 10, 15, 18-	
7	C7	Regular.Down.ActDay2.Modes5	8-, 9-, 15	5, 10, 12
8	C8	Regular.Down.ActDay2.Modes6	12, 15, 20-, 24	5-, 7-, 9-, 11
9	C9	Regular.Down.ActDay2.Modes7	2-, 8-, 10, 20-, 23-	2, 7-, 14-, 23, 24-
10	C10	Regular.Up.ActDay2.Modes1	7, 20, 24-	3, 5, 6, 9, 11-, 12-, 14-, 17-, 18
11	C11	Regular.Up.ActDay2.Modes2	1-, 8, 10-, 18	
12	C12	Regular.Up.ActDay2.Modes3	1-, 7, 10-, 20, 23-	8, 13-, 15-
13	C13	Regular.Up.ActDay2.Modes4	1-, 12-, 18, 24-	
14	C14	Regular.Up.ActDay2.Modes5	9-, 10-, 15-, 20, 24-	
15	C15	Regular.Up.ActDay2.Modes6	1, 12-, 18, 24-	1-, 2, 3, 4, 6, 7, 8, 14-, 16-, 17-, 18-, 19-, 20-, 21-, 22, 23
16	C16	Regular.Up.ActDay3.Modes1	15-, 18, 24-	
17	C17	Regular.Up.ActDay3.Modes2	1-, 10-, 15-, 18, 24-	19-
18	C18	Regular.Up.ActDay3.Modes3	2-, 10, 15-, 18, 24-	1, 2, 3-, 6-, 11, 13, 15, 16, 17, 18, 19, 20, 21, 22-
19	C19	Regular.Up.ActDay4.Modes1	15-, 17-, 18, 24-	24
20	C20	Regular.Up.ActDay4.Modes2	1, 2-, 15-, 18, 23-	3-, 4, 5, 8-, 9-, 10, 12, 14
21	C21	Regular.Up.ActDay4.Modes3	15-, 18, 24-	
22	C22	Regular.Up.ActDay4.Modes4	2, 15, 18-, 24-	
23	C23	Regular.Up.ActDay5.Modes1	1, 9, 15, 23-	4, 9-, 12-, 20-, 23-, 24-
24	C24	Regular.Up.ActDay5.Modes3	4, 9-, 19, 23-	2-, 3, 8, 10-, 13-, 14-, 15-, 16-, 17-, 18-, 19-, 21-, 22-

Table H: Inward and outward connections of the reconstructed gene regulatory networks for the **lymph node** compartment. Negative signs in these tables indicate negative coefficients in the ODE model.

	ID	Cluster.Name	Inward	Outward
1	C1	Regular.Down.ActDay1.Modes1	5, 10, 11, 15-, 16	2, 3, 4, 5-, 6, 8, 9, 10-, 11-, 12-, 13-, 14-, 15-, 16-
2	C2	Regular.Down.ActDay2.Modes0	1, 2, 11, 16	2, 4, 6, 7, 8, 9, 11, 12-, 14-, 15, 16-
3	C3	Regular.Down.ActDay2.Modes1	1, 3, 10, 11, 16	3, 5
4	C4	Regular.Down.ActDay2.Modes2	1, 2, 10, 11-, 16	11
5	C5	Regular.Down.ActDay2.Modes3	1-, 3, 5, 16	1, 5, 6-, 7, 9-, 14
6	C6	Regular.Down.ActDay3.Modes0	1, 2, 5-, 11, 16	
7	C7	Regular.Down.ActDay3.Modes1	2, 5, 10-, 11, 16	9
8	C8	Regular.Down.ActDay3.Modes2	1, 2, 11, 15, 16	
9	C9	Regular.Down.ActDay4.Modes0	1, 2, 5-, 7, 16	10, 13
10	C10	Regular.Up.ActDay1.Modes1	1-, 9, 10-, 11, 12-	1, 3, 4, 7-, 10-, 11-, 12-, 15-
11	C11	Regular.Up.ActDay2.Modes0	1-, 2, 4, 10-, 11	1, 2, 3, 4-, 6, 7, 8, 10, 11, 12-, 13-, 14-, 15, 16-
12	C12	Regular.Up.ActDay2.Modes1	1-, 2-, 10-, 11-, 16-	10-
13	C13	Regular.Up.ActDay2.Modes2	1-, 9, 11-, 14, 16-	
14	C14	Regular.Up.ActDay3.Modes0	1-, 2-, 5, 11-, 16-	13
15	C15	Regular.Up.ActDay3.Modes1	1-, 2, 10-, 11, 16-	1-, 8, 16-
16	C16	Regular.Up.ActDay3.Modes2	1-, 2-, 11-, 15-, 16-	1, 2, 3, 4, 5, 6, 7, 8, 9, 12-, 13-, 14-, 15-, 16-

Table I: Inward and outward connections of the reconstructed gene regulatory networks for the **spleen** compartment. Negative signs in these tables indicate negative coefficients in the ODE model.

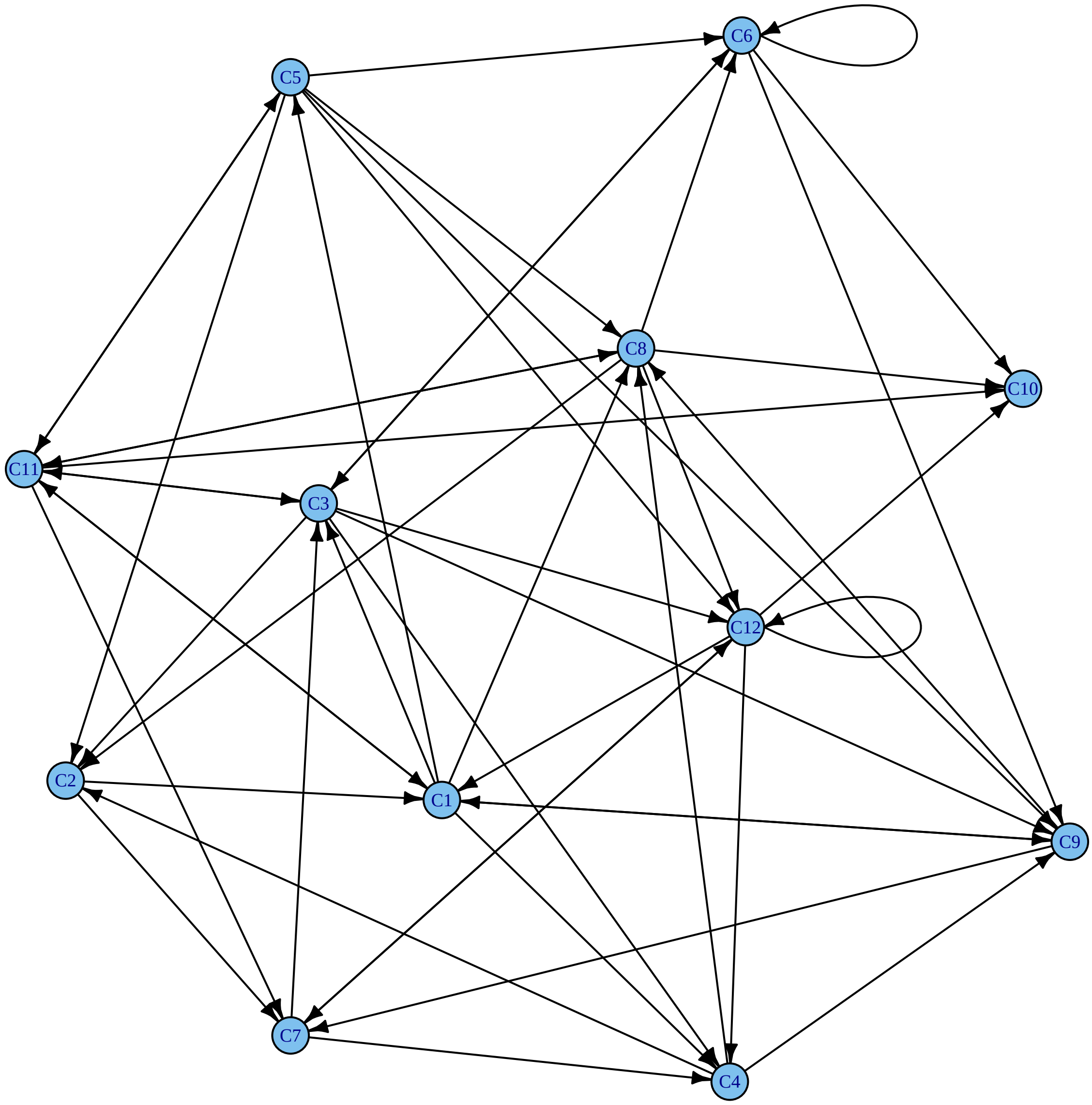

Figure L: **Network built from the blood compartment.**

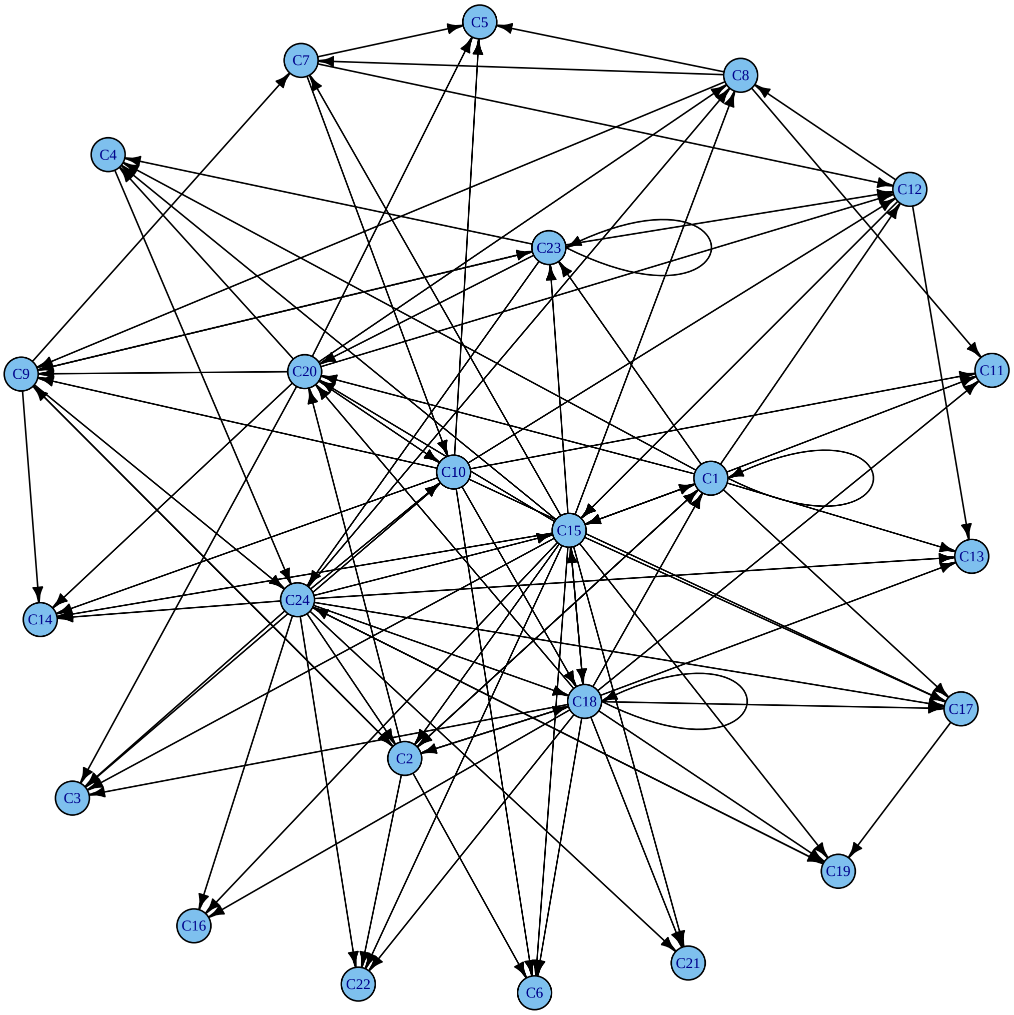

Figure M: **Network built from the lymph node compartment.**

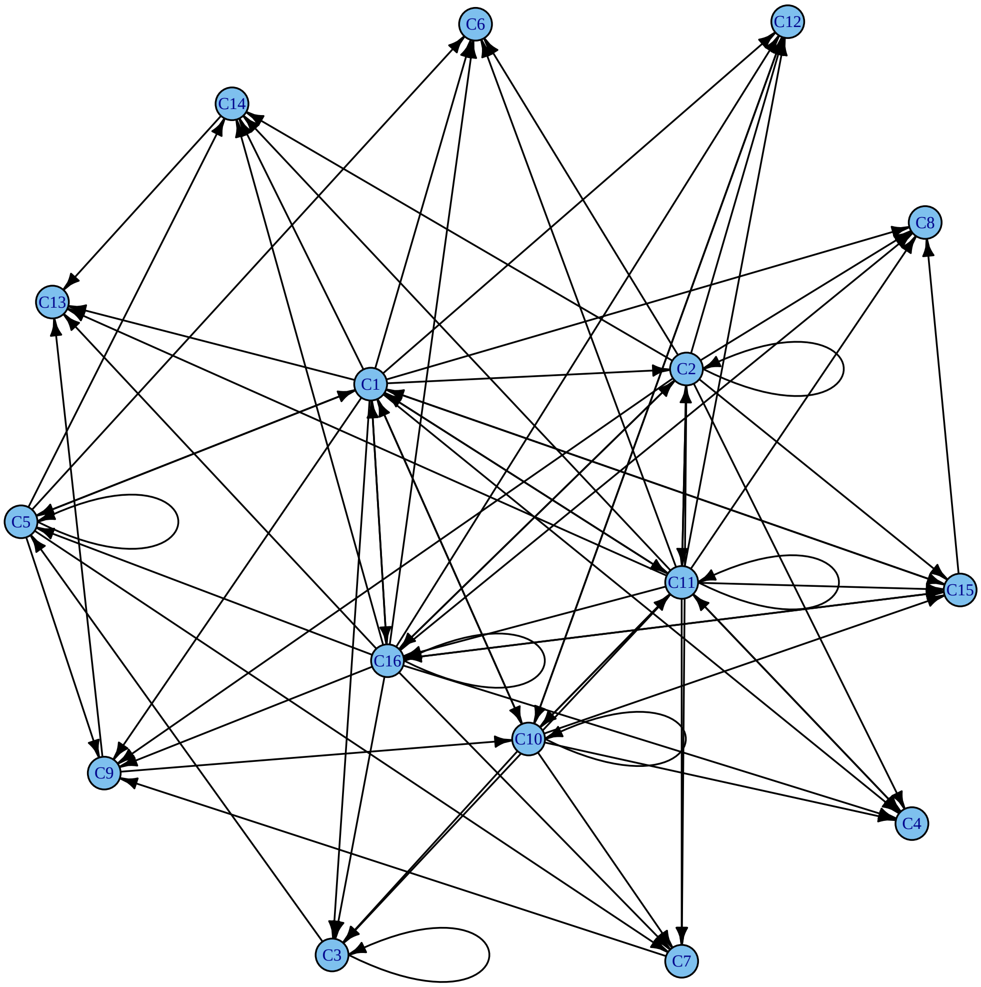

Figure N: **Network built from the spleen compartment.**

Buffer effects

In the lung compartment, 36 samples were stored in two different buffers, one contains 23 samples and the other 13 samples, before sending for microarray analysis. A quality assurance analysis revealed that samples from different buffers can have very different temporal pattern. Consequently, all 13 samples stored in the smaller buffer were excluded from the final analysis.

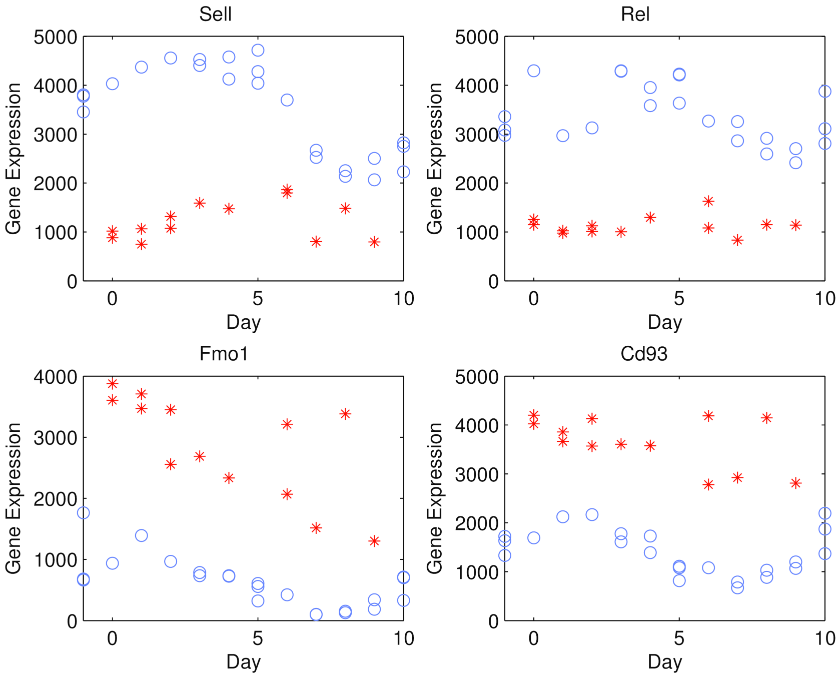

Figure O: **Four randomly selected genes showing buffer effects in data for the lung compartment. The red stars (13 samples in total) were removed from subsequent analyses.**

Eigen-functions of each compartment

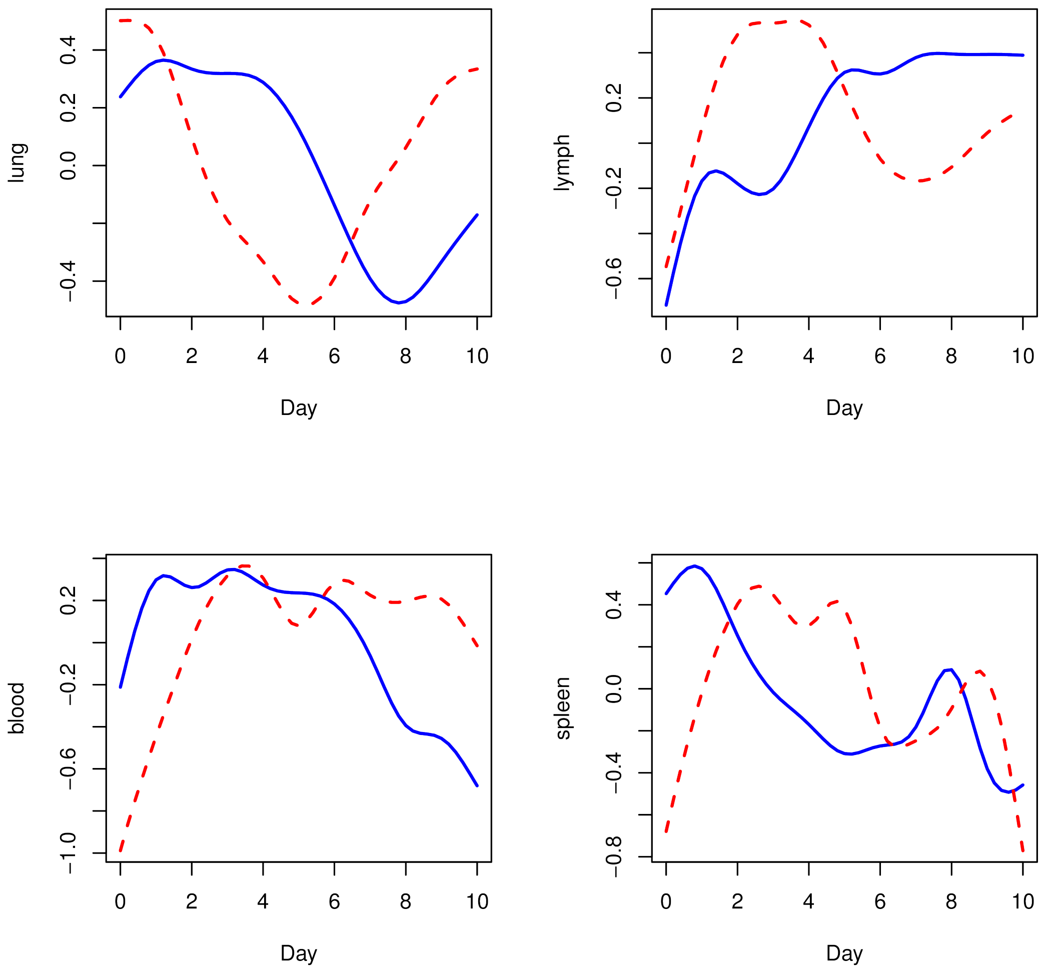

Figure P: **Leading eigen-functions in each compartment.**

Temporal trend of body-weight loss

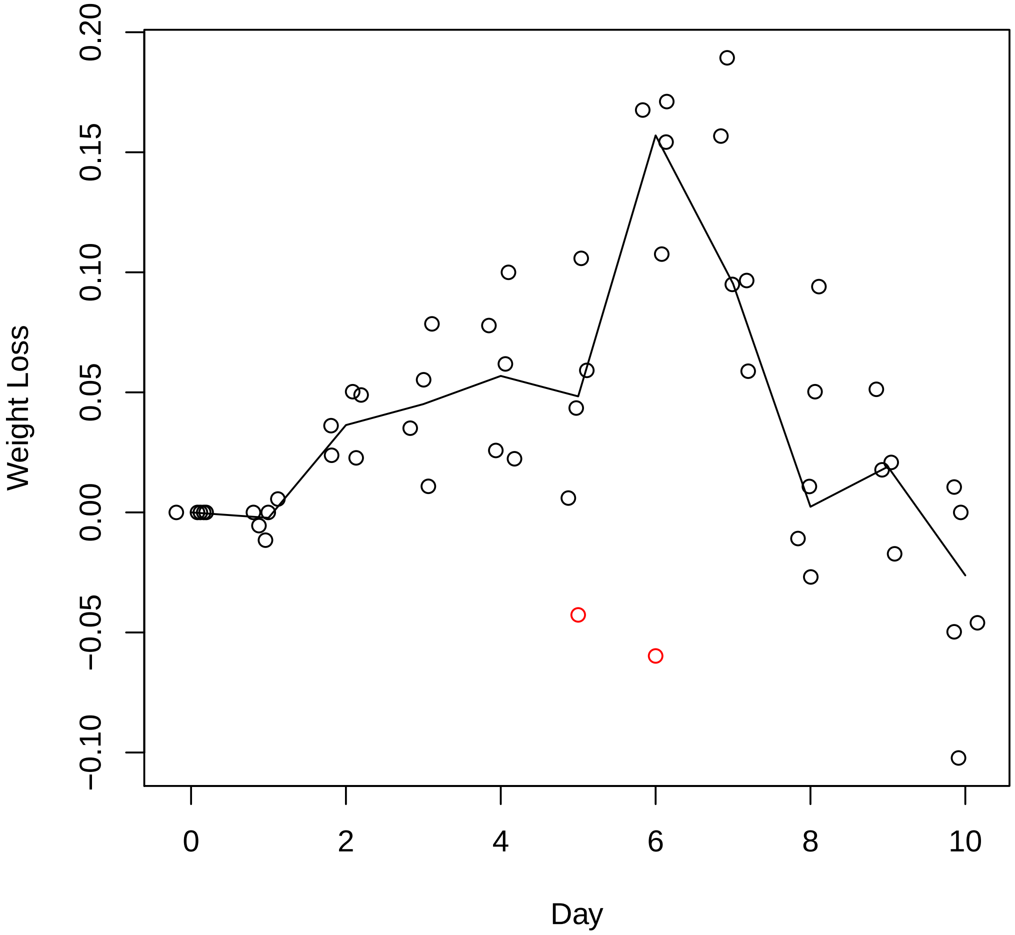

Figure Q: **Percentage of weight loss as a function of time. Weight loss peaked around day 6 post infection, with animals typically displaying around 15% weights loss as compared to Day 0 weights. Animals recovered all weight, and exhibited normal behaviour by Day 9 post infection. Two mice gained body weight on Day 5 and 6 post infection, respectively. These two outliers were marked by red color in this figure. The DPI coordinates are jittered slightly for better visual effects. The mean trend (solid black curve) displayed in this figure is computed from locally weighted scatterplot smoothing method (LOWESS).**

Temporal trend of the expression levels of notable inflammatory genes

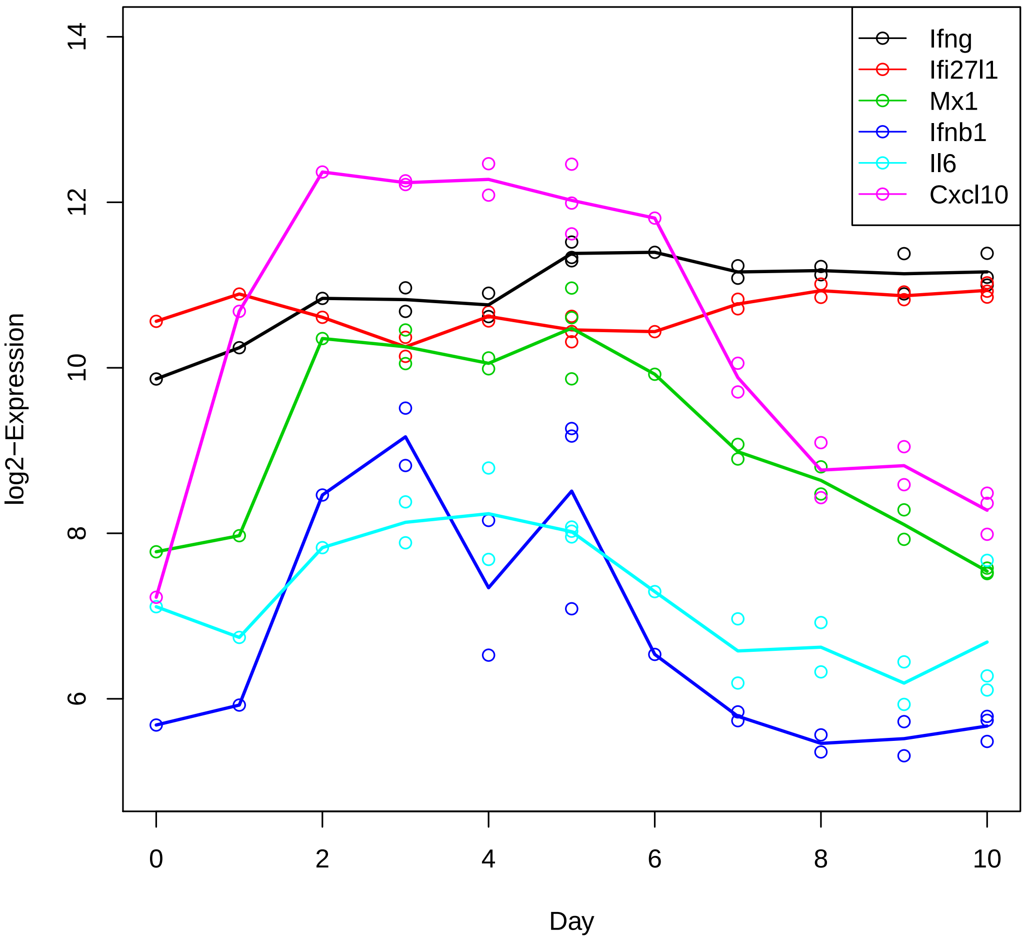

Figure R: **Gene expression as a function of DPI in the lung compartment.**
